# Accelerating transmission capacity expansion by using advanced conductors in existing right-of-way

**DOI:** 10.1073/pnas.2411207121

**Published:** 2024-09-23

**Authors:** Emilia Chojkiewicz, Umed Paliwal, Nikit Abhyankar, Casey Baker, Ric O’Connell, Duncan Callaway, Amol Phadke

**Affiliations:** ^a^Goldman School of Public Policy, University of California, Berkeley, CA 94720; ^b^GridLab, Berkeley, CA 94704; ^c^Energy and Resources Group, University of California, Berkeley, CA 94720

**Keywords:** power systems, decarbonization, transmission, renewable energy

## Abstract

As countries pursue decarbonization goals, the rapid expansion of transmission capacity for renewable energy (RE) integration poses a significant challenge due to hurdles such as permitting and cost allocation. However, we find that large-scale reconductoring with advanced composite-core conductors can cost-effectively double transmission capacity within existing right-of-way, with limited additional permitting. This strategy unlocks a high availability of increasingly economically viable RE resources in close proximity to the existing network. We implement reconductoring in a model of the US power system, showing that reconductoring can help meet over 80% of the new interzonal transmission needed to reach over 90% clean electricity by 2035 given restrictions on greenfield transmission build-out. With $180 billion in system cost savings by 2050, reconductoring presents a cost-effective and time-efficient, yet underutilized, opportunity to accelerate global transmission expansion.

## Significance

The integration of renewable energy sources at speed and scale in order to reduce emissions and achieve climate goals will likewise require the increase of transmission capacity at speed and scale. While the build-out of new greenfield lines is often plagued by challenges related to permitting and cost allocation, leveraging existing right-of-way, particularly through reconductoring with advanced conductors, can rapidly expand transmission capacity. However, advanced conductors have been traditionally viewed as a niche solution and their deployment is limited, requiring targeted policy to spur uptake and unlock their potential to contribute to cost-effective decarbonization.

Increasingly, the energy transition discourse is focusing on electricity transmission: the need to build it and the challenges of doing so. The International Energy Agency estimates that the global length of transmission lines must increase from 5.5 million to 15 million km—approximately 2.7 times—to reach net zero emissions by 2050, not including the eventual replacement of aging infrastructure (1). In the United States and Europe, however, new overhead lines take an average of over 10 y to build (1, 2). Grids are increasingly becoming the bottleneck of the energy transition, with over 1,200 GW of renewable energy (RE) projects in the United States, and over 3,000 GW globally, awaiting connection to the grid (3, 4). Challenges related to permitting—such as securing new right-of-way (ROW), completing environmental impact assessments, and cost allocation—often result in project delays (1, 2). In the United States, for example, the rate of transmission build-out has fallen by nearly 50 percent over the past decade, threatening decarbonization timelines (5, 6).

Recent rapid declines in the costs of solar, wind, and batteries (7) along with incentives from the Inflation Reduction Act (IRA) have presented an opportunity for a paradigm shift in how transmission is planned and sited. Specifically, there is a narrowing gap in cost between RE sited at locations with the highest resource potential and RE sited at locations that are in close proximity to the existing transmission network and load. This RE capacity could be unlocked through a wide range of technological solutions that can increase the transmission capacity of the existing grid. Some strategies, known under the umbrella term of Grid-Enhancing Technologies (GETs) and including Power Flow Controllers, Flexible AC Transmission Systems devices, Dynamic Line Ratings (DLR), and demand-side measures, can either enhance the physical capability of a transmission asset or the efficiency of power flow throughout the system. However, while these technologies are extremely important to expanding grid capacity, their potential is dependent on real-time operating conditions and thus typically limited and temporary. Other strategies can provide a larger and lasting increase of transmission capacity, such as reconductoring with advanced composite-core conductors, voltage upgrades, and AC-to-DC conversion. Yet whereas voltage upgrades may necessitate widening of the existing ROW and AC-to-DC conversion is generally most suitable for long lines, reconductoring—the replacement of a transmission line’s existing conductors with either larger-diameter conductors or a different type of conductor—is a practice used by utilities to increase ampacity within existing ROW.

**Fig. 1.**
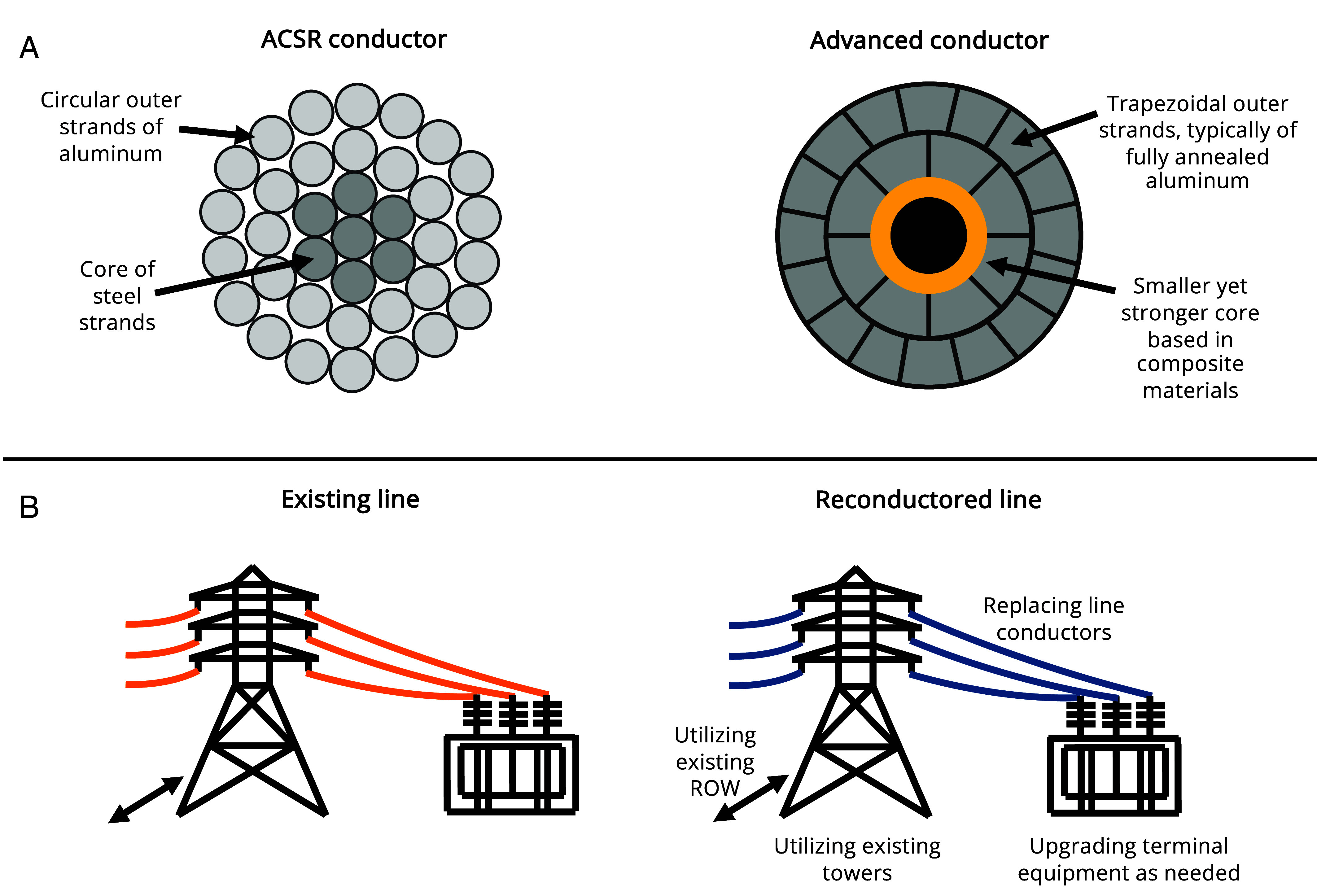
Conventional conductor technology compared to lines reconductored with advanced conductors. (*A*) A comparison of a cross-section of a conventional ACSR conductor compared to an equivalent-diameter advanced composite-core conductor (for more details, see *SI Appendix*) and (*B*) a schematic of an existing transmission line reconductored with advanced composite-core conductors.

In recent decades, the development of advanced composite-core conductors has opened up new possibilities for rapid transmission capacity expansion through reconductoring (8). While most of the high voltage grid today is wired with a century-old technology known as Aluminum Conductor Steel Reinforced (ACSR) featuring aluminum strands around a steel core (9), advanced conductors swap the steel for a stronger yet smaller composite-based core. This enables higher operating temperatures and more conductive aluminum to fit within an equivalent diameter, allowing advanced conductors to carry approximately twice as much power over ACSR (Fig. 1*A*). The composite-based core also reduces line sag, meaning the utilization of advanced conductors in reconductoring projects minimizes the need for and thus the costs of modifying structures to accommodate preexisting clearances, as reconductoring with conventional high-ampacity conductors such as Aluminum Conductor Steel Supported may risk larger sags. Because reconductoring projects leverage existing transmission towers and ROW, the extensive land acquisition and permitting processes that impede the construction of new lines can be circumvented (Fig. 1*B*) (8–11).

Previous work has established that it is cost-effective and time efficient to expand transmission capacity by reconductoring existing lines (8–12). Further, advanced conductors may offer additional advantages such as reduced galvanic corrosion and lower line losses during certain operating conditions (*SI Appendix*, Figs. S1 and S2 and Table S1). Over 90,000 miles of advanced conductors have been deployed globally (see *SI Appendix* for case studies), and manufacturing is widespread, including 3M, Southwire, CTC Global, TS Conductor, and Epsilon (13–17). However, in the United States, the technology is generally regarded as a niche solution for large spans such as river crossings. Further, major US power system planning studies (18–23), models (24), and existing planning tools (25) limit analysis to the construction of new lines only, or omit the most widely deployed composite-core conductor to date, CTC Global’s Aluminum Conductor Composite Core (ACCC) (10, 26). While the selection of the technological solution to increase transmission capacity should be carefully evaluated based on project needs, technical parameters, costs, timeline constraints, grid topology, and environmental conditions, these apparent advantages support the investigation of reconductoring with advanced conductors. However, no study has investigated the transmission capacity expansion potential of reconductoring at scale.

In this article, we show how recent developments have converged to present an opportunity for large-scale reconductoring to enable rapid transmission expansion US-wide. We first assess the transmission capacity increase and associated cost to reconductor all 53,000 US transmission lines. We select the most widely deployed composite-core conductor to date—CTC Global’s ACCC—for evaluation, although many other advanced conductors with similar thermal capabilities are available. We implement the resulting unit cost estimates in a widely used transmission and generation capacity expansion model, the Regional Energy Deployment System (ReEDs) (24). We apply constraints on the rate of transmission build-out to capture the permitting and cost allocation challenges that delay the development of transmission projects. Our modeling shows that reconductoring enables nearly four times as much transmission capacity to be added between the 134 ReEDS zones by 2035 at a marginally higher investment cost, compared to the case when only greenfield expansion is allowed at the recent historical rate. Reconductoring unlocks a high availability of cost-effective renewable resources in close proximity to the existing US transmission network and load, helping to meet over 80% of the new interzonal transmission needed to reach over 90% clean electricity given restrictions on greenfield transmission build-out. We also find that reconductoring can be a promising solution for intrazonal transmission capacity expansion, given that these lines tend to be shorter with lower unit costs to reconductor. These results indicate that reconductoring should constitute a key pillar in strategies to achieve grid decarbonization goals.

## Results

### Capacity Increases and Costs.

**Fig. 2.**
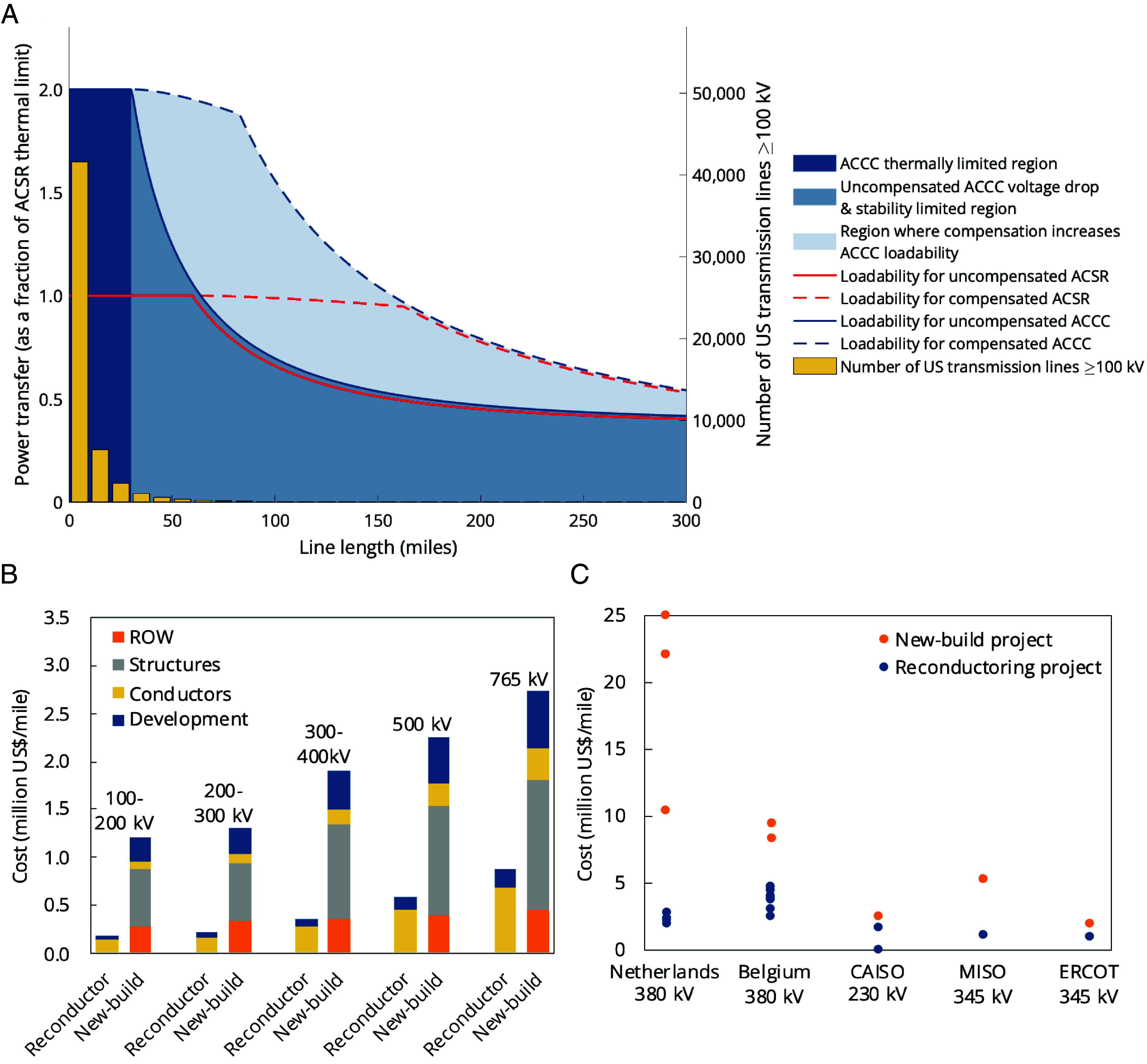
Capacity increases and costs of reconductoring. (*A*) The St. Clair’s curve for ACSR and ACCC conductors represents a piecewise measure of transmission line loadability as a function of line length, with the governing constraint—i.e., the thermal, voltage drop and angular stability limits—defining each interval of the curve. A full system study including load flow, contingency, and dynamic stability analyses should be conducted to verify these numbers in each real-world system. (*B*) Bottom–up cost estimates for reconductoring projects and new-build projects by voltage level (*Methods*). Estimates for new-build projects with ACSR are in line with generic estimates from other popular transmission planning tools (24–26), falling within 20% for each voltage level. (*C*) Empirical project cost data from Europe and the United States, presented by jurisdiction since cost definition and composition may vary (*SI Appendix*, Table S2) (31–39).

Reconductoring with advanced composite-core conductors raises the line conductor’s thermal limit, improving its ability to withstand higher temperatures of operation without compromising its structural integrity. We show this in the St. Clair’s curves in Fig. 2*A*, which plot line loadability of ACCC and ACSR lines as a function of line length; assuming the base case line is wired with ACSR and the reconductored line is wired with an equivalent-diameter ACCC, reconductoring with voltage support as needed can raise the line’s thermal limit and double transmission capacity for lines up to approximately 50 miles, comprising 98% of existing transmission segments in the United States today (27–30). Meanwhile, 2% of US alternating current (AC) transmission segments are above 50 miles (30), with their rated transfer capacity likely constrained by nonthermal factors such as voltage drop and/or angular stability limits. To fully reap the benefit of increased thermal capacity offered by reconductoring, these voltage drop and stability limits can be improved with additional voltage support in the form of reactive power compensation and/or sectionalization [the addition of new substation(s) with active and reactive power generation sources along the line, see *SI Appendix*, Figs. S3 and S4]. Sectionalization at most every 50 miles can shorten the effective line length, and thus with voltage support as needed, can similarly up to double transmission capacity (*SI Appendix*) (Fig. 2*A*).

We estimate the bottom–up cost to increase transmission capacity through reconductoring projects vis-a-vis new-build projects with ACSR (Fig. 2*B*). Although advanced conductors currently cost two to four times more than conventional conductors on a unit length basis due to higher raw material costs and limited scale of production (9, 11), the total cost of reconductoring projects on a unit length basis is less than half of new-build projects due to the avoided cost of new ROW and structures (*SI Appendix*). These findings are reflected in empirical cost data from reconductoring and new-build projects in Europe and the United States (Fig. 2*C* and *SI Appendix*, Table S2).

### Role in Capacity Expansion.

To demonstrate the utility of reconductoring to achieving decarbonization goals, we extend the ReEDS model to include reconductoring as a decision variable. We first calculate the cost to reconductor each of the 53,000 transmission lines in the United States (defined as a segment at or above 100 kV) (30) based on voltage level and line length (*Methods* and *SI Appendix*, Table S3). Like other power system planning models that require tractability and computational efficiency to draw insights into transmission needs (18–22), ReEDS simplifies the real-world system into 134 zones connected by 300+ transmission paths. We estimate the cost of reconductoring each ReEDS path by taking a GW-mile weighted average of the cost to reconductor each individual line that makes up the path, use these per-line costs to generate a supply curve for the path, then run a least-cost system optimization investigating system expansion under four scenarios: with and without reconductoring as an option, and with and without constraints on the rate of transmission build-out, on a time horizon up to 2050. We consider IRA incentives and increases in load corresponding to high electrification. Furthermore, reflecting pending policy from the Environmental Protection Agency (EPA), and consistent with a net-zero pathway, we model the phase-out of coal generation by 2035, and we block the construction of new gas-fired capacity (for more details, see *Methods*). For new-build lines, build-out constraints reflect permitting and cost allocation challenges through nationwide, interregional, and intraregional constraints based on recent historical rates; for reconductoring projects, build-out constraints reflect cost allocation challenges for interregional lines through a similar constraint based on recent historical rates (for more details, see *Methods*).

**Fig. 3.**
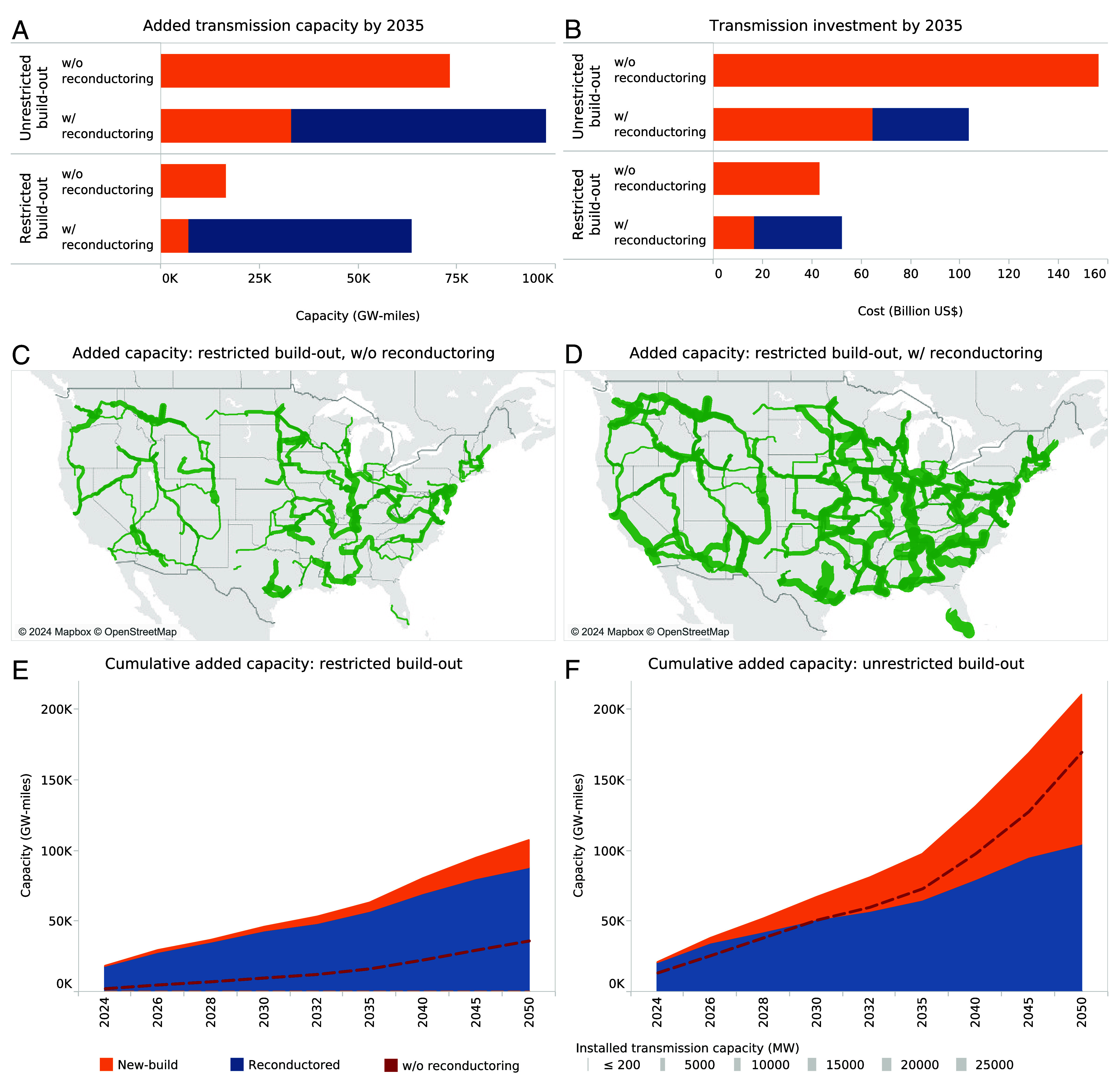
Added interzonal transmission capacity and associated investment. Added interzonal transmission capacity (*A*) and total interzonal transmission investment (*B*) between 2022 and 2035, by scenario, in 2022 US$. Transmission investment includes both line as well as substation costs; reconductoring projects are conservatively assumed to require a new substation. We also show added interzonal transmission capacity between 2022 and 2035 for the restricted build-out scenario by ReEDS path, without reconductoring as an option (*C*) and with reconductoring as an option (*D*), as well as the cumulative interzonal transmission capacity build-out with reconductoring for the restricted case (*E*) and the unrestricted case (*F*) over time.

We find that when reconductoring is an option, it is favored over building new lines due to its lower cost, representing 66% of interzonal transmission capacity added by 2035 in the unrestricted build-out case (Fig. 3*A*). This indicates that even without factoring in the benefit of faster project realization resulting from leveraging existing ROW, reconductoring should be considered as a key strategy for expanding transmission capacity purely based on its cost competitiveness. The significance of reconductoring is even more pronounced in the case where build-out is restricted to the recent historical rate, enabling nearly four times as much new interzonal transmission capacity to be added by 2035 at only slightly higher total investment cost compared to the case with only new-build (Fig. 3 *A* and *B*). The resulting transmission capacity increase with reconductoring is therefore not only larger but also distributed over more transmission corridors (Fig. 3 *C* and *D*). Further, regardless of build-out rate restrictions, reconductored capacity accounts for the majority of interzonal capacity added before 2030 (Fig. 3 *E* and *F*). Although this trend is likely driven by the lower cost of reconductoring, considering that new lines often take 10 to 15 y to complete (1, 2, 4), reconductoring presents a synergistic opportunity for expanding transmission capacity in the near-term while new lines are planned and permitted. We additionally find that the average intrazonal line length is considerably shorter than interzonal (7 miles compared to 30 miles) and that the average unit cost of reconductoring intrazonal transmission lines is about 20% lower than interzonal lines, making a compelling case for the reconductoring of intrazonal transmission lines as well.

The larger and more distributed interzonal transmission capacity increase enabled by reconductoring simultaneously unlocks access to lower-cost, higher-quality RE in more locations (*SI Appendix*, Fig. S5). The combined effect of lower transmission expansion costs and higher-quality RE lowers total generation and transmission costs by 3 to 4% (*SI Appendix*, Fig. S6), translating to $85 billion in system cost savings by 2035 and $180 billion by 2050. This is notable considering the fact that although we do not impose a constraint to reach a certain clean energy share by a certain year, all four scenarios reach over 90% clean energy by 2035, and correspondingly commensurate greenhouse gas emissions, largely due to low clean energy costs resulting from IRA incentives and the absence of conventional fossil-fuel alternatives. The system cost savings unlocked by reconductoring are largely a result of the variation in which technologies are installed to meet load across the four scenarios (*SI Appendix*, Fig. S7). In the restricted build-out case and without reconductoring as an option, the model relies more heavily on an expensive technology not currently available at scale—gas-fired generation with carbon capture and storage—which is consistent with other studies (5, 19). This indicates that large-scale reconductoring can facilitate the cost-effective achievement of decarbonization goals while also mitigating the risk and uncertainty that comes with the development of transmission requiring new ROW, the siting of renewable projects, and the commercialization of dispatchable zero-carbon technologies.

**Fig. 4.**
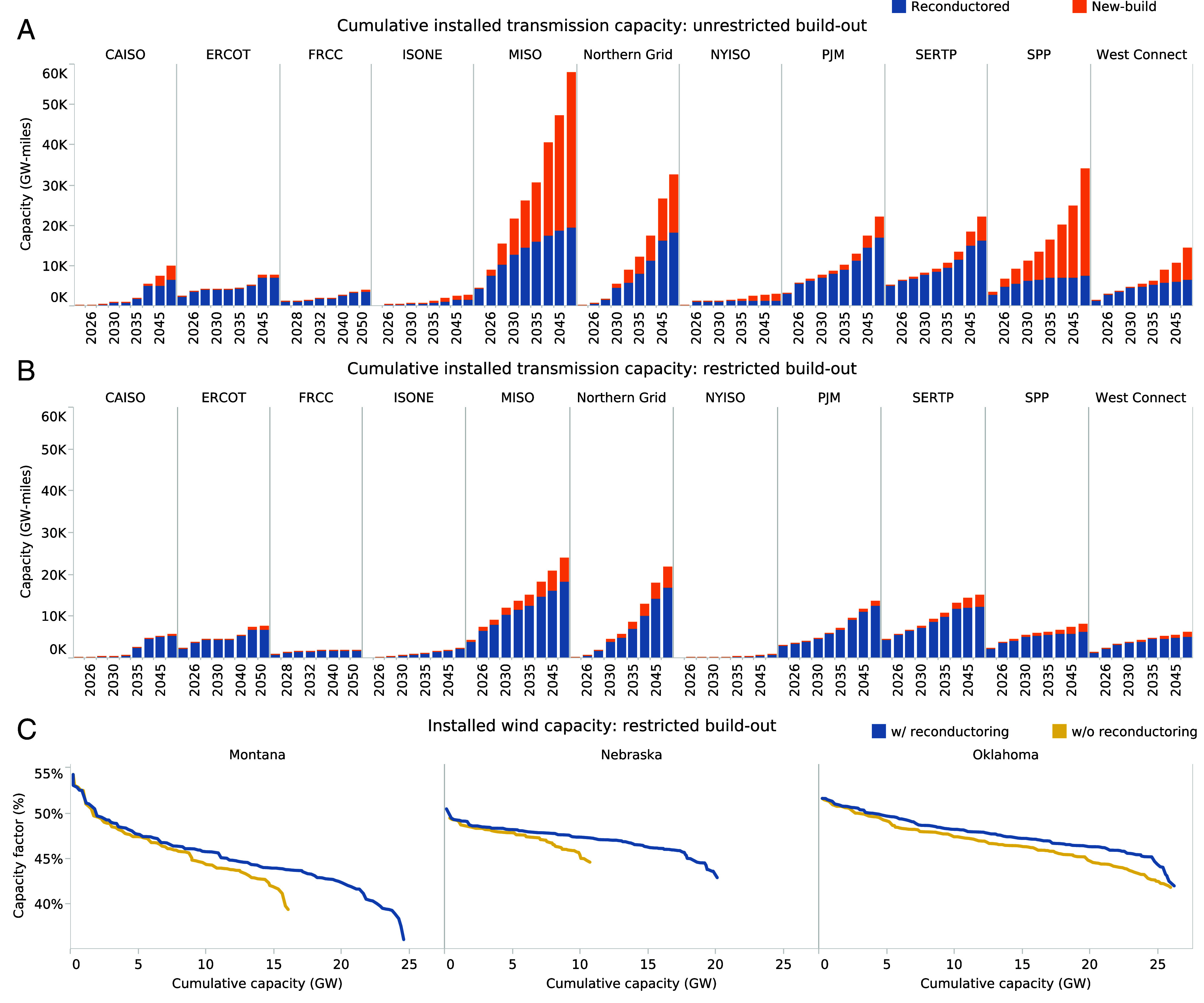
Regional variation in transmission capacity expansion. Cumulative interzonal transmission capacity for the unrestricted build-out scenario (*A*) and the restricted build-out scenario (*B*). We also show the capacity factor of installed wind farms as a function of the cumulative installed wind capacity for the restricted build-out case (*C*).

Given that transmission expansion needs and their respective barriers may vary widely by planning region, we analyze the added interzonal transmission capacity over time by transmission planning region (Fig. 4 *A* and *B* and *SI Appendix*, Fig. S8). In regions such as the Electric Reliability Council of Texas (ERCOT), Pennsylvania-New Jersey-Maryland Interconnection (PJM), and California ISO (CAISO), reconductoring comprises a larger share of the total added interzonal transmission capacity compared to regions such as the Midcontinent Independent System Operator (MISO) or Southwest Power Pool (SPP) (Fig. 4 *A* and *B*). In these latter regions, significantly more new-build interzonal transmission capacity is added in the unrestricted build-out (Fig. 4*A*) case than in restricted build-out case (Fig. 4*B*), in order to access prime onshore wind resources—the least-cost RE resource—within the central parts of the United States. Even in the restricted build-out case, reconductoring enables more wind capacity to be accessed in wind-rich states, as demonstrated by Montana and Nebraska, and at higher capacity factors, as demonstrated by Oklahoma (Fig. 4*C*). The trend holds for other wind-rich states such as Idaho and Illinois, although notably does not hold for the wind-rich state of Texas, where high-quality wind resources cannot be accessed due to limited cross-interconnect capacity with neighboring states.

## Discussion

The timely build-out of transmission capacity is key to integrating the RE resources necessary for meeting decarbonization goals. However, the commercial availability of composite-reinforced advanced conductors with high-temperature and low-sag capabilities has created an opportunity to meet a majority of near-term transmission needs through leveraging existing ROW. Our results indicate that reconductoring can rapidly and cost-effectively increase transmission capacity and unlock RE on a US-wide scale, contributing to over 80% of the new interzonal transmission needed to reach over 90% clean electricity by 2035 given restrictions on greenfield transmission build-out. This informs optimal investment decisions and demonstrates the importance of a holistic system planning approach that jointly considers generation and transmission investments.

Increasing transmission capacity may offer additional notable yet difficult-to-quantify advantages. Previous work has noted that although today’s approach to transmission planning focuses primarily on reliability benefits (40), transmission build-out also importantly helps reduce congestion and mitigate extreme grid conditions through improved resiliency and interregional trade (41–44). Reconductoring can help support these benefits—especially in light of the clogged interconnection queue (3, 4), high uncertainty about load and variable generation forecasts (45), and the increasing frequency and severity of extreme weather events (46, 47)—given that it enables a larger and more distributed increase of transmission capacity. While the reconductoring process may involve taking the line out of service while work is completed, which can pose a challenge in already-congested networks, the work can be performed circuit-by-circuit in seasons of low demand and in applicable cases while the line remains energized (see *SI Appendix* for case studies). Further, from an operational perspective, the elimination of the steel core in composite-core conductors has been demonstrated to significantly improve corrosion resistance compared to conventional ACSR (48), and a reconductoring project can enable real-time monitoring, DLR as well as improved wildfire protection through the inclusion of a fiber-optic cable within the conductor. The evaluation of these many potential benefits should be incorporated into transmission planning processes.

Some regions are capitalizing on this opportunity more than others. For example, both the Netherlands and Belgium have decided to reconductor most of their high-voltage backbone by 2035. Through detailed transmission network modeling, these countries’ system operators have identified reconductoring as the fastest and most cost-effective strategy to support rapid RE integration, reduce congestion, and overcome difficulties in securing new ROW (31–33). The adoption of innovative, efficiency-based solutions—like advanced conductors, but also DLR and topology control, among others—has been encouraged by the European Union as well as on a national level through a variety of policies, that authorize public funding, accelerate project permitting, and offer innovation incentives (49–53). Similarly, the transmission planning philosophy in India—where demands of rapid load growth necessitate strategies that increase the capacity of both transmission and distribution systems in a limited time frame—dictates the optimization of ROW utilization, specifying reconductoring of existing AC transmission lines with higher ampacity conductors as one example (54). Projects are increasingly evaluated on a total cost of ownership basis rather than the conventional capex estimation, with the inclusion of an ohmic loss evaluation in many project tenders that favors advanced conductors’ lower resistance, resulting in India boasting some of the largest deployment rates of advanced conductors in the world (55, 56).

Policymakers and regulators in the United States need to consider similar options. The Montana State Legislature recently passed a law establishing cost-effectiveness criteria for advanced conductors (57), and other states should follow suit. Meanwhile, the DOE or IEEE could consider a national conductor efficiency and/or resistance-based standard—similar to the energy conservation standards for distribution transformers—to ensure that advanced conductors make their way into widespread use (58). Further, because reconductoring has the potential to unlock RE capacity and accelerate transmission capacity expansion on a large scale, the strategy’s benefits cannot be fully captured by evaluating its merits solely on a line-by-line basis, motivating the consideration of reconductoring within system planning processes. Federal Energy Regulatory Commission (FERC)’s most recent reform of transmission planning, Order 1920, mandates the evaluation of advanced conductors and reconductoring practices as alternatives to new lines within long-term regional transmission planning, ensuring reconductoring is considered in ways similar to DLRs and advanced power flow control devices (59). The DOE’s Grid Deployment Office could also identify opportunities for reconductoring within the National Transmission Needs Study (20), while the Loan Programs Office could conduct outreach with utilities to garner proposals for reconductoring projects. Utilities themselves can solicit grant proposals under the Bipartisan Infrastructure Law’s Smart Grid Grants program. Meanwhile, outreach to Independent System Operators (ISOs), Regional Transmission Operators (RTOs), state regulators, and other advocates can help quantify the opportunity and compel transmission builders and owners to embrace this technological solution.

Transmission networks are complex, and the actual increase in power transfer capacity offered by reconductoring is determined by a multitude of factors beyond the scope of this analysis. We recommend that transmission owners, ISOs, and RTOs perform more detailed project-level assessments—including load flow, contingency, and dynamic stability analyses—to evaluate the wide-scale deployment of advanced conductors and more broadly consider the array of commercially available solutions that can increase power density in their existing networks with regard to their technical parameters, costs, project needs, timeline constraints, grid topology, and environmental conditions. This includes pairing reconductoring projects with line voltage increases or other GETs, particularly if large capacity increases are desired. Project-level assessments would also better capture terminal equipment upgrade needs, such as transformers and protection equipment; here, we assumed that reconductoring projects require a new substation, whereas in practice substations may be upgradeable at lower cost. While we study reconductoring with an equivalent-diameter advanced conductor, even higher thermal capacity increases are possible by reconductoring with an equivalent-weight advanced conductor and/or including different coatings. Further, the reconductoring of lower-voltage lines may simultaneously increase the rated capacity of neighboring higher-voltage lines that may be constrained by stability or contingency limits. A reconductoring project may also provide an opportunity to simultaneously perform maintenance work – such as reinforcing existing towers or replacing insulators—which may be motivated by the existing infrastructure’s age and condition. However, because we assume advanced conductors are the same or less weight as the existing conductor, and operated at the same voltage, this maintenance work would not be a direct requirement of the reconductoring effort and thus its costs should be allocated separately. Moreover, sectionalization with inverter-based resources and grid-forming inverters appears to be an emerging and promising strategy (60) to integrating renewable generation and support system stability through reactive power support, inertia, frequency response, and black start capability (61), yet additional technical assessment is needed to realize mass deployment in bulk power systems. Future work is planned to explore the potential transmission capacity increase of other technological solutions that can increase the transmission capacity of the existing grid (like AC-to-DC conversion and DLR); conduct power flow analysis and investigate system stability implications of reconductoring and sectionalization to understand the benefits of coordinated transmission and resource planning; and investigate the potential for large-scale reconductoring in other global regions.

## Methods

### Estimate the Capacity of Existing Lines.

We obtain data on US transmission lines from the US Homeland Infrastructure Foundation-Level Data (HIFLD) (30), at 100 kV and above as per the methodology of National Renewable Energy Laboratory (NREL)’s ReEDS model (24). For each voltage, we define the surge impedance in Ohms (taking the upper limit as a conservative value) to estimate the surge impedance loading (SIL), which aligns with other estimates (29, 62, 63):

$$\text{SIL}\;[\text{MW}]=\frac{(\text{Voltage}\;[\text{kV}]{)}^{2}}{\text{Surge}\;\text{Impedance}\;[\text{Ohms}]}.$$

We use these SIL values to estimate the rated capacity for each line utilizing the standard St. Clair’s curve defining line loadability as a function of distance to obtain a length-dependent SIL multiplier (29). This multiplier applies to all voltage levels except for 765 kV, where the thermal limit is defined as 2.7*SIL for line lengths up to 50 miles as per MISO’s safe loading limits (62). As technical line configuration is unknown, we assume one circuit per line, no installed compensation, and that ratings are constant throughout the year (i.e., no seasonal ratings). The resulting total estimated transmission capacity (~190 TW-miles) falls within other estimates of the current TW-miles deployed in the United States (150 to 200 TW-miles) (5, 21, 24).

### Estimate the Capacity of Reconductored Lines.

Given the limitations of the standard St. Clair’s curve for calculating loadability with advanced conductors and/or varying compensation, we analytically derive St. Clair’s curves for an equivalent-diameter ACSR and ACCC line; we consider both zero and unlimited compensation at the receiving end and assume the ACCC conductor’s thermal limit is 2× and resistance is 0.75× that of the ACSR conductor (13, 16, 27, 28). We extend the St. Clair’s curves up to 300 miles, the length of the longest AC transmission line in the United States (30). From these curves, we quantify the capacity increase through reconductoring—based on the ratio of ACCC loadability over uncompensated ACSR loadability—and determine the set of complementary strategies that is used based on the line length. Lines between 0 and 30 miles do not require any other complementary strategy as they fall within the thermal limit; lines between 30 and 50 miles can leverage voltage support to enable a doubling of line capacity with reconductoring, with the quantity of reactive power compensation determined by theory from refs. 27, 28; and for the 2% of US transmission lines above 50 miles, sectionalization [the addition of new substation(s) with active and reactive power generation sources along the line, likely with a grid-forming inverter] at most every 50 miles can shorten the effective line length that with voltage support as needed can similarly up to double transmission capacity. In line with previous St. Clair’s curve derivations, we assume the curves hold across varying voltage levels, though some minor differences may occur for example due to conductor size and configuration (29). However, with the exception of resistance, conductor properties like reactance and susceptance remain the same across different types of conductors with the same diameter (64).

### Estimate the Cost to Reconductor Existing Lines.

In Fig. 2*B*, we build up the generic costs of expanding a line’s transmission capacity through reconductoring and compare it with the conventional approach of building a new line parallel to the existing ROW, consisting of the ROW, structures, conductors, and development.

#### ROW.

Since reconductoring projects take place within existing ROW, no new land is required. For new lines, we utilize the US-average cost of pasture land from the US Department of Agriculture; although land costs may vary widely by state and be significantly elevated especially in urban or suburban areas (39, 65). Although a new line that runs parallel to an existing ROW may be able to utilize some or all of an already-secured ROW, this may not always be the case and we conservatively assume that an entirely new ROW must be secured based on ROW width by voltage level (*SI Appendix*, Table S3) (25). To the land costs, we add acquisition costs along with regulatory and permitting costs (39).

#### Structures.

We assume all new structures are steel towers and include the costs of materials, installation, hardware, and the structure foundation for the various structure types (tangent structures, running angle structures, nonangled deadend structures, and angled deadend structures) and their respective quantity per mile approximations (39). Reconductoring does not typically require any structure modification so structure costs are assumed to be zero, although other necessary maintenance work is often performed concurrently with the reconductoring.

#### Conductors.

We estimate the costs of the conductors based on the material, installation, and accessories costs of ACSR and equivalent-diameter ACCC® conductors (39). For each voltage level, we establish a reference conductor size selection and bundle quantity (*SI Appendix*, Table S3) (25, 39). We assume a sag and wastage adder of 4% to the conductor material costs (39). For new lines, we assume that a shield wire is necessary for each circuit (39). For reconductoring, we assume that the aluminum from the former ACSR conductor can be recovered and recycled—at 50% the 5-y average price of new aluminum—which is then subtracted from the total costs (66).

#### Development.

For development, we assume a contingency of 10%, a 5.5% project management adder, a 1.5% administrative overhead adder, and a 3% engineering, testing, and commissioning adder, added to the sum of the ROW, structure, and conductor costs (39). We also assume a 7% adder for the allowance for funds used during construction, added to the sum of the ROW, structure, conductor, and contingency costs (39). We do not include terrain multipliers because the HIFLD dataset of US transmission lines does not contain sufficient information on the terrain for each segment (30), and there is no concrete evidence on the varying labor/installation costs resulting from varying terrain.

#### AC Terminals.

The upgrades to AC terminal stations within a reconductoring project are heavily dependent on the ratings of the existing terminal equipment, most notably the transformers and protection equipment. The ReEDS model accounts for terminal costs separate from line costs, so we use the provided terminal costs in ReEDS for both reconductoring and new-build lines, conservatively assuming reconductoring projects require an entirely new substation. For lines with an effective length of 30 to 50 miles, we do include the cost of voltage support within the reconductoring line cost based on the costs of a static var compensator, representing the median cost among various compensation technologies (39), with the quantity of compensation determined by theory from refs. 27, 28. For lines above 50 miles, we also include the cost of sectionalization at most every 50 miles within the reconductoring line cost—reflecting a new 6-position (double-breaker bus) substation (39)—although these costs are typically allocated to the generators that are seeking access to the transmission system.

To this generic cost build-up, we add the cost of compensation and sectionalization, as a function of voltage level and line length, to estimate the total cost in US$/mile to reconductor each of the ~53,000 transmission lines in the United States at 100 kV and above (30). We then incorporate the previously quantified delta capacity increase to obtain unit costs in US$/MW-mile.

### ReEDS Model Setup.

We utilize the ReEDS capacity expansion and dispatch model from the NREL for the contiguous US electric power system, in order to assess the impacts of reconductoring on future generation capacity additions, electricity costs, new transmission development, etc., by 2050 on a national scale (24). The ReEDS model was chosen over other capacity expansion models for its open-source nature, spatiotemporal granularity across the contiguous United States, and extensive use in US resource planning studies such as the National Transmission Needs Study (20). The model utilizes a system-wide least-cost optimization approach to identify the most cost-effective mix of electricity generation, storage, and transmission technologies that can meet electric power demand. This optimization takes into account factors such as grid reliability, technology resource constraints, and policy constraints, and is performed in 2-y intervals starting from 2010, with the capability to extend simulations up to the year 2100. The model yields a range of key outputs including generator capacity, annual generation from each technology, storage and transmission capacity expansion, total sector costs, electricity prices, as well as fuel demand, prices, and CO_2_ emissions. Although ReEDS can also simulate the power sectors of Canada and Mexico, it is primarily focused on the contiguous United States, dividing the country into 134 model balancing areas that are interconnected by approximately 300 representative transmission paths, thereby providing a granular geographical and regulatory representation.

We use the 2022 version of ReEDS in this study which includes all the state and federal policies as of December 2022, including both the recently passed IRA and the Infrastructure Investment and Jobs Act. We include stringent site exclusions as per the reV model (67). We model a high rate of electrification, with correspondingly high load and high zero-carbon generation build-out. To the base model, we also add additional constraints to retire coal capacity by 2035, implemented linearly with the oldest plants retiring first, and disallow new gas capacity post-2023, except for plants that are already under construction. This reflects pending policy from the EPA that seeks to strengthen emission limits and guidelines for carbon dioxide from fossil fuel-fired power plants, along with the investment uncertainty regarding the construction of new fossil fuel-fired power plants. No additional transmission capacity expansion is allowed between the three interconnects (East, West, ERCOT) nor across national borders (Canada, Mexico).

### Implementing Reconductoring in ReEDS.

The ReEDS model represents transmission via a synthetic network of 134 nodes connected by 300+ transmission paths, based on the real-world grid. The capacity of each path is determined from power flow analysis, incorporating individual line ratings. Meanwhile, the nodes are generally located in the center of each zone, also known as a balancing area. While this means that the ReEDS model inherently focuses on the build-out of interzonal transmission rather than intrazonal transmission or spur lines, power system planning studies of large systems like the contiguous United States must generally scale down the existing transmission system into a synthetic model for tractability and computational efficiency; however, these studies still draw broader conclusions about transmission needs (18–22). We match every physical transmission line with a path in ReEDS and estimate its cost of reconductoring by taking a GW-mile weighted average of the cost to reconductor each individual line that makes up the path and use these per-line costs to generate a supply curve for the path. While this approach is considered sufficient for the scope of this study—in order to quantify the nationwide potential for reconductoring and its role in facilitating the integration of RE resources—it is important for transmission owners, ISOs, and RTOs to perform more detailed studies with their system planning models to evaluate the wide-scale deployment of advanced conductors in their systems.

By default, ReEDS only allows new-build transmission expansion, whose costs per MW-mile are calculated based on the voltage level of existing lines within the balancing area with regional multipliers. To model the option of reconductoring in the ReEDS model we provide a supply curve, composed of two bins with costs for each path: the first bin being reconductoring, capped at double the path’s existing capacity in ReEDS, and the second bin being new-build capacity requiring new ROW, with unlimited build-out potential.

### Modeling Transmission Constraints in ReEDS.

For the restricted build-out scenarios, we represent permitting and cost allocation challenges through the addition of several constraints. For new-build lines that are potentially hindered by both these issues, we limit the total nationwide expansion to 1,400 GW-miles/y, the 2010 to 2021 average rate (6, 24). For new-build lines, we also apply intraregional and interregional constraints, limiting annual expansion to the recent intraregional and interregional rates, respectively, for each region. For reconductoring, which may be hindered by interregional cost allocation issues, we similarly limit annual expansion of interregional capacity to the recent interregional rate. For the purposes of this study, transmission “region” refers to the FERC Order No. 1000 transmission planning regions and includes the CAISO, ColumbiaGrid, Florida Reliability Coordinating Council, ISO New England, Midcontinent ISO (MISO), Northern Grid, New York ISO, PJM, WestConnect, SPP, ERCOT, South Carolina Regional Transmission Planning, and Southeastern Regional Transmission Planning.

## Supplementary Material

Appendix 01 (PDF)

## Data Availability

Key modeling results and indicative transmission corridors that could benefit from capacity increases to unlock renewables can be explored in ArcGIS (68). All data needed to evaluate the conclusions in the paper are included in the paper and/or *SI Appendix*.
