# Supplementary material for: Accelerating transmission capacity expansion by using advanced conductors in existing right-of-way
Source: Proc Natl Acad Sci U S A. 2024 Sep 23;121(40):e2411207121. doi: 10.1073/pnas.2411207121 (PMC11459140; doi:10.1073/pnas.2411207121)
Supplement: Supplementary file 1 — Appendix 01 (PDF) [file pnas.2411207121.sapp.pdf]

PNAS

Supporting Information for

Accelerating transmission capacity expansion by using advanced conductors in existing right-of-way

Authors: Emilia Chojkiewicz[1], Umed Paliwal[1], Nikit Abhyankar[1], Casey Baker[2], Ric O'Connell[2], Duncan Callaway[3]*, Amol Phadke[1]**

Author Affiliations:

[1]Goldman School of Public Policy, University of California, Berkeley; Berkeley, California 94720, USA

[2]GridLab; Berkeley, California 94704, USA

[3]Energy and Resources Group, University of California, Berkeley; Berkeley, California 94720, USA

*Co-corresponding Author. Email: dcal@berkeley.edu

**Co-corresponding Author. Email: aaphadke@berkeley.edu

This PDF file includes:

Supporting text:

1. Background information on advanced conductors
2. Real-world reconductoring project case studies
3. Impacts of sectionalization on power system stability

Figures S1 to S8

Tables S1 to S3

SI References

Supporting text

1. Background information on advanced conductors

The most common conductor utilized for overhead high voltage power transmission in the US today is the Aluminum Conductor Steel Reinforced (ACSR), featuring conductive aluminum strands around a supporting steel core *(1,2)*. Its many advantages like good conductivity, low weight, low cost, utilization of common materials and resistance to corrosion cemented its place as the industry standard since it was invented in the early 1900s to this day. However, lines wired with ACSR are typically technically limited to normal operating temperatures of approximately 75°C *(2)*, above which the tensile strength decreases over time, weakening the conductor and increasing susceptibility to failure.

Despite numerous efforts to improve conductor design over ensuing decades, enhancements in one aspect of conductor design often led to trade-offs with other features. This is reflected by the example of the Aluminum Conductor Steel Supported (ACSS), first introduced in the 1970s. By utilizing aluminum strands that were fully annealed, the conductor could withstand higher operating temperatures and thus increased power transfer capacity. However, operation at higher temperatures ran the risk of excessive sag due to the high thermal expansion of steel; this drawback limited the benefits of ACSS, necessitating taller structures or shorter spans in new lines to accommodate prescribed minimum clearances and/or under-rating reconductored lines below their thermal capacity to abide by the pre-existing sag restriction *(3,4)*.

In recent years, advancements in materials science have given rise to advanced conductors which can carry approximately up to twice the current, and thus double the power, of conventional conductors. Also known as high temperature low sag (HTLS) conductors, they swap the conventional steel core for a smaller and lighter composite core (typically ceramic, glass or carbon fibers) without compromising structural strength. Therefore, more aluminum - typically annealed aluminum, as it has the highest temperature capabilities - can fit within an equivalent diameter and higher operating temperatures can be achieved, all while abiding by the sag restrictions that often limit a line's rated capacity (*4-8*). In contrast to conventional conductors, the technology behind composite-based conductor cores is typically proprietary, with several US companies leading the market: examples include the Aluminum Conductor Composite Reinforced (ACCR) made by 3M, the Aluminum Conductor Composite Core (ACCC) made by CTC Global, and the Aluminum Encapsulated Carbon Core (AECC) made by TS Conductor *(5-8)*. These manufacturers typically produce the conductor cores in-house, then packaged on reels and shipped to vendors which strand the aluminum strands around the core and supply the ready conductor to end-users. Table S1 compares the technical characteristics of these advanced conductors with ACSR and ACSS.

Advanced conductors have undergone significant laboratory testing by relevant institutions such as the Electric Power Research Institute (EPRI), the International Council on Large Electric Systems (CIGRE) and others with their work publicly available in technical reports and brochures. Results indicate that other advantages of advanced conductors may include improved resistance and resilience to bending failure, oxidation, UV waves, galvanic corrosion, and general environmental damage, varying by conductor model *(1,8-10)*. Some advanced

conductors also embed an optical fiber to monitor line temperature and elongation in real time, enabling validation testing after installation, dynamic line ratings, and/or insulation-based wildfire protection. Furthermore, their installation typically follows similar procedures and tools as for ACSR, avoiding the need for special training and/or equipment.

Another result of advanced conductors' higher aluminum content and/or use of annealed aluminum, their electrical resistance is about 20-30% lower than ACSR conductors; this improved conductivity in turn reduces I^2R or "copper" losses, with advanced conductors manufacturers claiming loss reductions of up to 50% *(5-8)*. For a utility, this can translate into considerable operational savings as well as emissions reductions through the offset of fossil fuel generation and/or increased savings of renewable generation, depending on the local resource mix (*10*). However, these claims are typically made under the assumption that the line loading stays the same, which would likely not apply to a reconductoring project where lines are upgraded with advanced conductors in order to increase their thermal carrying capacity.

We evaluate these claims by considering a "Drake"-size ACSR and ACCC conductor and using the IEEE Standard for Calculating the Current-Temperature of Bare Overhead Conductors *(11)*. The ACCC conductor operates at lower temperatures for equivalent current (Fig. S1A) due to the lower thermal conductivity of its composite-based, rather than steel-based, core. Likewise, although conductor resistance varies linearly with operating temperature (Fig. S1B), the ACCC conductor's resistance increases at a lower rate than the ACSR conductor for increasing operating temperature.

We next consider a double-circuit 345 kV line with 3 phases and 2 "Drake"-size conductors per phase. In the base case, the line is wired with ACSR. Grid planners have two options for increasing capacity through the corridor: reconductor with ACCC or build a new line with ACSR parallel to the existing one. We calculate line losses for the original case and the two upgraded cases via

$$Line\ losses\ =\left[\frac{I_\varphi}{N_{cond}}\right]^2\cdot R\cdot N_{cond}\cdot N_{circ}\cdot N_\varphi\cdot FLA$$

and calculate line load via

$$Line\ load\ =\sqrt{3}\cdot I_\varphi\cdot N_{circ}\cdot V$$

where I_φ is the phase current, N_{cond} is the number of conductors per phase, R is the resistance per unit length, N_{circ} is the number of circuits per line, N_φ is the number of phases, FLA is the full load adjustment, V is the line voltage *(12)*. Losses increase exponentially with the line loading, yet for equivalent amps an ACCC reconductoring project would lower losses by up to ~30% compared to the original line (Fig. S2). After the line is upgraded and loaded above the thermal limit of the original line, losses are still lower when reconductoring with ACCC rather than building a new parallel line with ACSR up to approximately the point of emergency operation. However, since losses are heavily dependent on the utilization of a line, the overall change in annual losses before and after upgrading will depend on how frequently the line is lightly vs heavily loaded.

2. Real-world reconductoring project case studies

Belgium

In Europe, where advanced conductors are often referred to as high temperature low sag (HTLS) conductors, Belgium has pioneered deployment. Since the first installation in 2009, the country's Transmission System Operator (TSO) Elia has undertaken a wide-scale project to reinforce the majority of their 380 kV backbone with HTLS conductors by the mid-2030s *(13)*. Most of the existing backbone consists of double-circuit lines wired with All Aluminum Alloy Conductor (AAAC), rated at approximately 2000 A *(14,15)*. Beginning with the most congested lines, reconductoring will double the load transfer capacity to approximately 4000-5000 A, predominantly using the Aluminum Conductor Composite Core (ACCC) from CTC Global/Lamifil *(13-15)*. Motivating factors for grid reinforcement include the need to integrate renewables and energy storage, accommodate the geo-spatial shift of generation from retiring nuclear power plants to offshore wind resources in the North Sea, and support both domestic and industrial electrification (Elia predicts annual consumption to increase by up to 50%, from 80 to 120 TWh, between 2022-2032) *(16)*. The main reasons for using HTLS conductors over new corridors is their significantly faster realization, bypassing permitting delays and difficulties to secure new rights-of-way (ROW) due to high population density, as well as significantly lower capex; reconductoring projects take less than half the time and are less than half the cost of new-build projects.

Given Belgium's location in the heart of Europe and therefore frequent subjection to transiting power flows, reconductoring projects within and across its borders are also recognized for their potential to facilitate increased power trade and provide resiliency benefits to the greater continental grid. Interconnection projects in particular, given their increased cost-benefit ratios over building out new corridors, may be prioritized by the European Commission as a Project of Common Interest (PCI) and thus eligible to receive public funds and accelerated permitting *(17)*. They are then coordinated by the European Network of Transmission System Operators for Electricity (ENTSO-E) under the Ten Year Network Development Plan (TYNDP) framework, ensuring harmonized transmission planning across the continent *(17)*. Elia was also a key contributor of the European Commission's BEST PATHS project (Beyond State-of-the-art Technologies for rePowering AC Corridors and Multi-Terminal HVDC Systems), which from 2014 to 2019 expanded European TSOs' knowledge around the safe construction with and operation of HTLS conductors *(18)*.

A supportive regulatory ecosystem has likewise helped foster the widespread adoption of advanced conductors. The EU electricity market directive of 2009 directed regulators to grant system operators "appropriate incentive over both the short and long term, to increase efficiencies, foster market integration… and support the related research activities" *(19)*. In its implementation of this EU directive, Belgium expressly recognized the strategic importance of technical innovation in the electricity sector in their own law, and the Belgian regulator CREG (Commission for Electricity and Gas Regulation) worked closely with Elia to pursue the uptake of more innovative technologies *(20)*. The first reconductoring projects in the early 2010s took several steps to mitigate risks, which included demonstrating to authorities that safe clearances would be maintained under different operating conditions as well as the utilization of different

advanced conductors, complete with each supplier's corresponding accessories, on separate circuits in case of unforeseen technical problems *(13-15)*. Beginning in the 2016, CREG also introduced an "innovation incentive", which has led Elia to pursue other innovation and efficiency-based solutions, like digitalization, dynamic line ratings for overhead lines, and the installation of phase shifting transformers to better regulate power flows *(20)*. HTLS conductors are also viewed as a more sustainable solution over conventional conductors, given their composite-based cores do not use steel and thus have lower resistances, translating to lower line losses and improved efficiencies.

Netherlands

Like Belgium, Dutch TSO TenneT also plans to upgrade most of their 380 kV backbone to HTLS conductors for a load transfer capacity increase from about 2500 A to 4000 A *(21,22)*. Known as the "Beter Benutten Bestaande 380 kV" (Making Better Use of the 380 kV Grid) project, the first phase involves upgrading 191 km (119 miles) of transmission lines between 2019-2026 and the second phase plans to upgrade an additional 165 km (103 miles) by 2035 *(21,22)*. Motivating factors include difficulty to secure new rights-of-way (ROW) due to high population density, the need to rapidly integrate more renewable energy (RE) and in particular offshore wind resources, and the reduction of congestion enabling increased cross-border power trade.

In the Netherlands, the challenge of structural congestion in the high voltage transmission grid is particularly acute. Some areas of the grid have seen rapid electrification of industrial processes saturating spare transmission capacity, while other areas have seen explosive growth in interconnection requests from renewable energy generators *(23)*. The latest EU electricity market regulation in 2019 recognized these issues, impelling Member States to review bidding zones and address capacity allocation and congestion management with efficient market-based solutions; it also directed TSOs to ensure that at least 70% of cross-border transmission capacity is offered for cross-zonal trade *(24)*. In response, the Dutch action plan identified reconductoring with HTLS conductors as a key strategy to alleviate congestion and increase thermal transmission capacity in the near-term, along with exploring dynamic line ratings and improving dispatch coordination *(25)*.

With most reconductoring projects completed within a few years of conception, the replacement process is rooted in standardized practices shared by Elia and TenneT. Since most of the pertinent lines are double-circuit, one circuit remains live while the other is de-energized and reconductored, typically planned to coincide with seasons of lower demand. Other necessary maintenance work - such as the replacement of insulators, ground wires, bird flight diverters or strengthening of towers and mast foundations to bring them to the latest construction standards - is often combined and performed concurrently with the reconductoring *(13,21,22)*. Furthermore, the reconductoring may be combined with phase number optimization, in order to avoid expanding the magnetic field zone.

Italy

Whereas reconductoring projects in Belgium and the Netherlands pertain to relatively short line lengths (i.e., <90 km or <50 miles), Italy presents a case of transmission capacity expansion over significantly longer distances and larger scale. Motivating factors include difficulty to secure new rights-of-way (ROW) due to high population density, strengthening network reliability and resilience, and the need to rapidly integrate more renewable energy (RE). At the end of January 2023, requests from renewable generators to connect to the high voltage grid had reached 340 GW; in comparison, Italy had 32 GW of installed capacity of wind and solar in 2019 and forecasts an installed capacity of 102 GW in 2030 *(26,27)*.

Terna, the Italian TSO, plans to invest 11 billion Euros in a Hypergrid network in order to double the exchange capacity between market zones in the country (from 16 GW to 30 GW) by the mid-2030s *(26,27)*. In addition to reconductoring several 380 kV lines with advanced conductors capable of high-temperature operation, the plan notably envisions a large, multi-terminal HVDC network across the country. New north-south power lines are planned as undersea HVDC cables, rather than conventional AC overhead lines, due to "the impossibility of overhead lines or the need for synergy/efficiency with existing projects" *(26)*. Several existing overhead AC lines are also set to be "modernized" and thereby converted to HVDC operation, raising the voltage to 500 kV DC from either 220 or 380 kV AC, enabling the bulk transport of renewable energy from southern generation centers to northern load centers (*27)*.

Texas

While mass deployment of advanced conductors and reconductoring practices in transmission planning is commonplace in several European countries, at the time that it was completed, the Lower Rio Grande Valley (LRGV) reconductoring project in southeastern Texas was the longest in the world. The project involved reconductoring the two single-circuit double-bundle 345 kV transmission lines that serve the LRGV, doubling transmission capacity with CTC Global's ACCC conductor (to a 1988/2426 MVA normal/emergency rating) *(28,29)*. Motivating factors included rapid population growth in the area and seasonal peak demands that exceeded previously modeled projections, leading to rolling blackouts during the south Texas Ice Storm of February 2011. Although the local utility - American Electric Power (AEP) - considered conventional solutions such as the construction of new lines, the risks of permitting delays associated with ROW acquisition was seen as a serious deterrent to this time-sensitive project *(28)*.

Given the need to meet reliability demands within a constrained timeframe, an energized reconductoring of the line emerged as the only option. Although this required the construction of temporary poles, the poles were placed within the existing right-of-way. Therefore the project did not require time-intensive permitting for new land acquisition and was approved the same day it was presented to ERCOT's Board of Directors in 2011 *(28,29)*. Ultimately, the $225 million project was completed in 2016, several months ahead of schedule and millions of dollars under-budget (*28-31*).

India

Many emerging economies - where the demands of rapid electrification and load growth necessitate the consideration of strategies that increase power transfer capacity in a limited timeframe - are also turning to reconductoring. In India, the transmission planning philosophy dictates the optimization of existing ROW and costs under a long-term perspective, particularly for constrained areas including urban centers and difficult terrain. Guidelines explicitly outline the application of smart grid technologies (including FACTS devices and phase-shifting transformers), upgrade of existing AC transmission lines to higher voltages, reconductoring of existing AC transmission lines with higher ampacity conductors, the use of multi-voltage level and multi-circuit transmission lines, as well as the use of HVDC transmission *(32)*.

As the manufacturer of the most widely deployed advanced conductor, as of 2021 CTC Global had completed over 180 projects in India, accounting for approximately 16% of the company's 1,100 total projects with ACCC *(33)*. The projects have spanned 23 Indian states, deploying 15,000 km (~9,300 miles) of conductor to over 30 customers, on voltage levels ranging from 22 kV to 400 kV *(33-35)*. India's utilization of advanced conductors also highlights the technology's ability to increase capacity of distribution systems, as advanced conductors played an important role in the Saubhagya Scheme to bring electricity to every household, particularly in rural areas (*35,36*). State utilities have also been prioritizing energy efficiency through the inclusion of an ohmic loss evaluation in their tenders, which favors advanced conductors like ACCC due to their lower losses over conventional conductors *(32)*. Nearly ⅓ of the capital city of Delhi has been upgraded to ACCC conductors to increase capacity and improve grid reliability and efficiency *(33)*.

India also has seen the widespread deployment of 3M's Aluminum Conductor Composite Reinforced (ACCR), for example, around the land-constrained city of Mumbai *(37)*. Increasingly, the planning approach aims to evaluate conductor investments on a total cost of ownership basis, rather than a conventional cost estimation process, to more accurately capture conductor benefits.

China

China is also active in its adoption of advanced conductors, where they are utilized both in reconductoring projects as well as new lines. Motivated by rapid economic growth that has precipitated increased demand for electricity, advanced conductors offer an opportunity to efficiently reach transmission growth objectives. As previously described, CTC Global is one of the most active advanced conductor manufacturers. Alongside its core production facilities in the United States, Paraguay and Indonesia, CTC Global partnered with the NARI group (a wholly-owned subsidiary of the State Grid Corporation of China, the largest electricity utility in the world) to open a manufacturing plant in China in 2014 *(38)*. The primary objective of the new plant is to produce core exclusively for the Chinese market, which sees approximately 50 billion US$ in transmission investment each year *(39)*. For example, the plant supplied 291 km (180 miles) of ACCC conductor for a critical grounding line at one of the AC/DC converter stations of the 3,300 km (2,050 miles) 1100 kV Zhundong-Huainan HVDC project, set to deliver 66 TWh annually to eastern China *(40)*.

3. Impacts of sectionalization on power system stability

Recent work *(41)* and ongoing real-world projects like CAISO's new Manning substation *(42)* and NV Energy's new Greenlink transmission line *(43)* suggest that sectionalization - the addition of a new substation(s) with active and reactive power generation sources along the transmission line, likely with a grid-forming inverter - can help enhance transmission performance while incorporating the necessary renewable resources along existing right-of-way (ROW). Here, we investigate the impacts of sectionalization on power system stability using a simple 3-bus, 230 kV system based on *(44)* with a few changes. We select branch 1-3, as the branch from primary generation source to load, for investigation. We assign bus 1 to be the slack bus, remove the load and generation source at bus 2 to better isolate the performance on branch 1-3, add a new generation source at bus 3 and make the impedance on each line the equivalent, forming the business-as-usual (BAU) case. If the length of branch 1-3 l_{13} is over 50 miles, i.e., past the thermally-limited region, we sectionalize the branch by adding new buses n with active and/or reactive power generation sources p_n and q_n at evenly spaced intervals. These intervals, at most, are 50 miles long ($50<l_{13}\leq100$ results in 1 new bus and 2 sections, $100<l_{13}\leq150$ results in 2 new buses and 3 sections, and so on). The costs of generation sources p_n and q_n are set to zero so that they are preferred by the model, representing renewable resources that are increasingly the most cost-effective source of electricity generation. Single line diagrams of these systems are shown in Figure S3.

Since sectionalizing may affect bus voltages and voltage drops, we employ a continuation power flow in Matpower *(45)* to determine steady state stability limits. The base case starts at a load of p^d_3=100 MW, increasing to a target case load of p^d_3=1000 MW; generation at bus 3 is not scaled, so that the increasing load comes from the slack bus. The continuation power flow uses a step size of 0.05, pseudo arc length parametrization, and is set to enforce active and reactive power generation limits. The process terminates when a branch flow limit is reached. We investigate cases with and without bisection, with and without reconductoring all lines of the system with advanced conductors, and with and without different amounts of active and reactive power injection at the load bus 3 as well as the new sectionalized buses. For each case, the branch flow limit is calculated based on theory from *(46-48)* based on the voltage level, line length, conductor type, and availability of compensation. In Figure S4, we show results in the form of load bus 3 voltages over continuation parameter λ, which is proportional to the bus 3 active power demand.

For short lines, reconductoring enables higher power transfers over branch 1-3 and thus higher active power consumption at bus 3. Due to the lower resistance of ACCC over conventional ACSR, the PV curve shifts to the right for an equivalent bus 3 voltage. For long lines, which are not thermally limited, reconductoring alone offers minimal benefit to the system. However, the utilization of the existing transmission system may be enhanced through the sectionalization of very long lines with appropriate quantities of active and/or reactive power injection, especially when combined with reconductoring with advanced conductors which raise the thermal limit of operation. As seen in Figure S4, sectionalization of long lines with reactive power support at both the load bus 3 and the new sectionalized buses improves voltage stability at the load bus 3 and increases power transfer over branch 1-3. For very long lines with several sections, the amount of reactive power injection at each new sectionalized bus increases with increased

distance from the slack bus 1. In these cases, sectionalization and the injection of active and reactive power can boost power flow on branch 1-3 and thus enable higher demand at bus 3, which is directly proportional to the continuation parameter λ. While here we only consider a small 3-bus system, preliminary findings indicate that sectionalization may help integrate this required renewable capacity, along with storage, while simultaneously enhancing the utilization of the existing transmission system

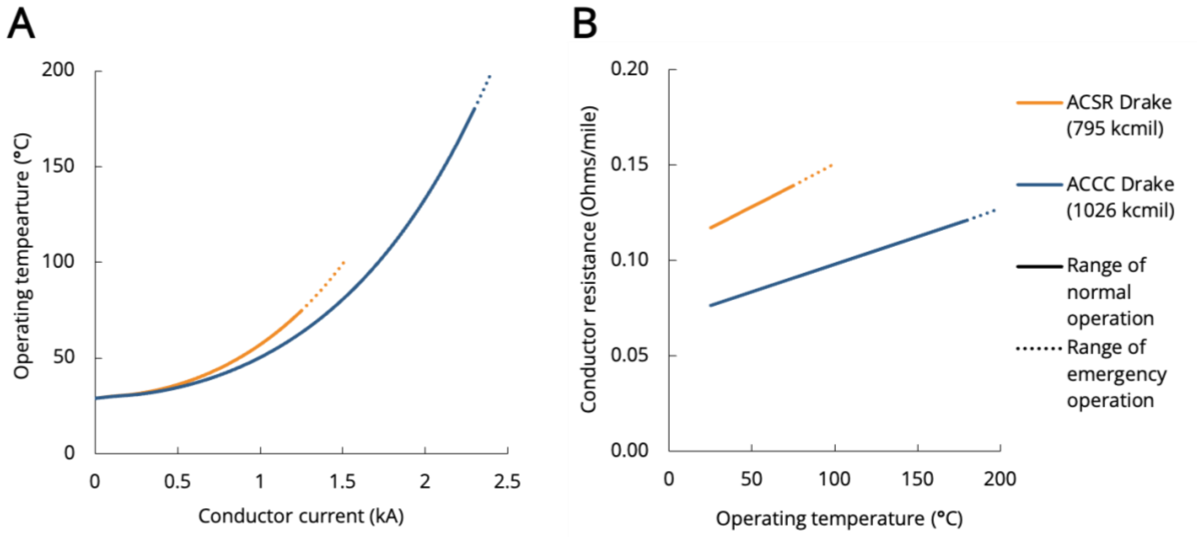

Figure S1: The relationship between conductor properties and operating temperature. (A) Conductor operating temperature as a function of current through the conductor. (B) Conductor resistance as a function of conductor operating temperature. Solid lines refer to the conductor's normal range of operation, while dotted lines indicate the conductor's range of emergency operation. Calculations performed at 25.0°C Ambient Temperature, 1028.7 (W/m²) Sun Radiation, 2.00 (m/s) Wind, 90 Wind angle, 0 (m) Elevation, 0.50 Solar Absorptivity, 0.50 Emissivity based on IEEE Standard 738-2006 for Calculating the Current-Temperature of Bare Overhead Conductors for a single "Drake"-size conductor *(11)*.

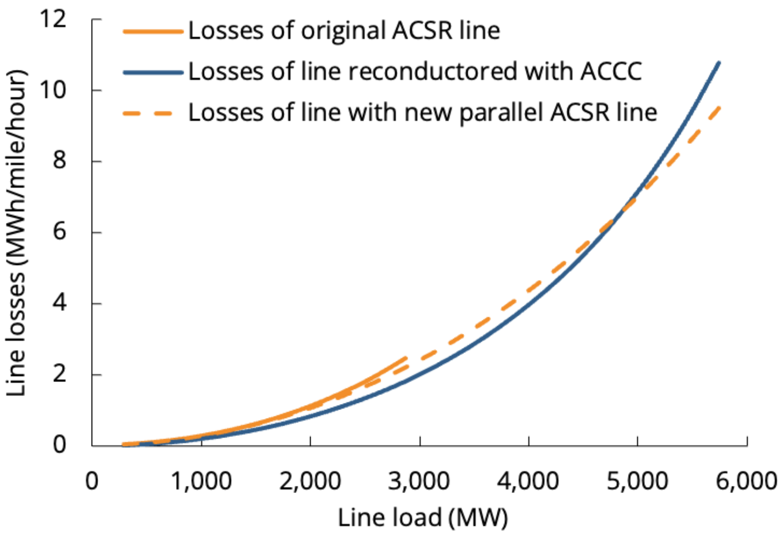

Figure S2: Line losses as a function of line load. The figure shows how the losses would change as a function of line loading for a double-circuit 345 kV line with 3 phases and 2 “Drake”-size conductors per phase, originally wired with ACSR, either through reconductoring or the building of a new parallel ACSR line.

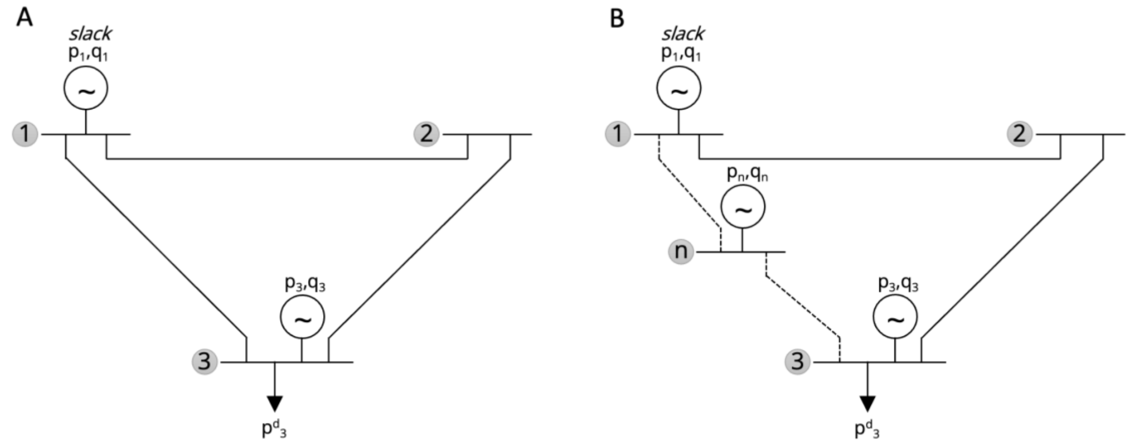

Fig. S3: Single line diagram of the three-bus test system. (A) The Business-As-Usual (BAU) case. (B) The sectionalized case, featuring a new substation between bus 1 and bus 3. In both cases, bus 1 serves as the slack bus while bus 3 serves as the load bus. The variables p_1 and q_1 refer to the active and reactive power at bus 1, respectively; p_3 and q_3 refer to the active and reactive power at bus 3, respectively; p_n and q_n refer to the active and reactive power at bus n (the sectionalized bus), respectively.

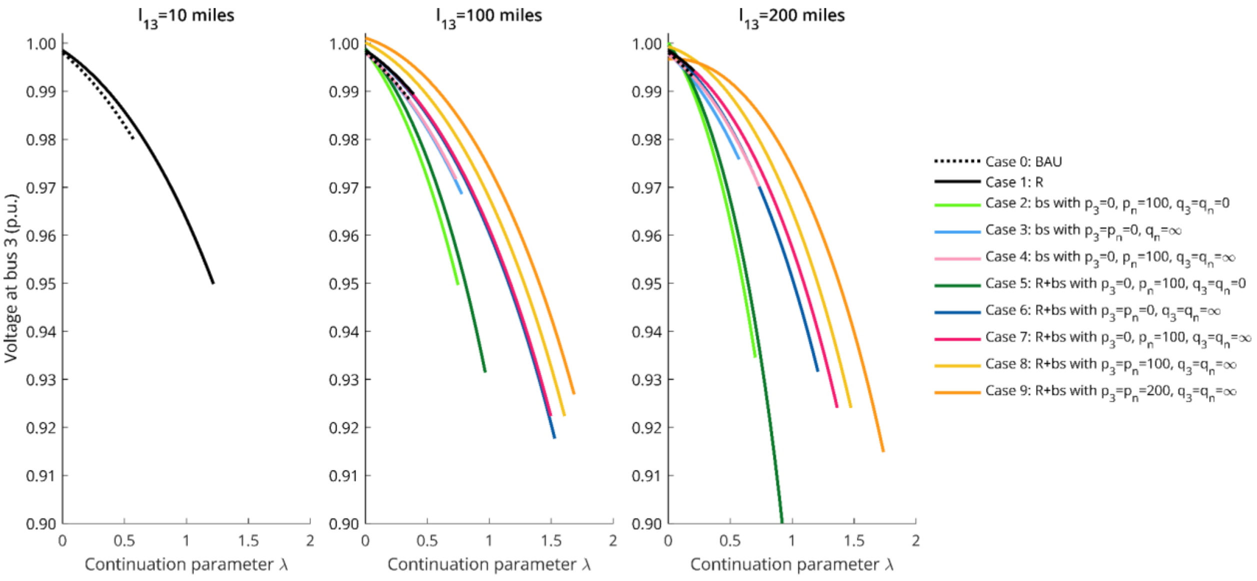

Fig. S4: PV curves of bus 3 for different lengths of branch 1-3. These PV curves show the results of the continuation power flow on various cases of reconductoring (R), bisection (bs), and reconductoring with bisection (R+bs) of the three-bus test system compared to Case 0, the Business-As-Usual (BAU) case. In long lines, sectionalization and the injection of active and reactive power can boost power flow on branch 1-3 and thus enable higher demand at bus 3, which is directly proportional to the continuation parameter λ. The variables in the legend refer to Fig. S3: p_3 and q_3 refer to the active and reactive power at bus 3, respectively; p_n and q_n refer to the active and reactive power at bus n (the sectionalized bus), respectively.

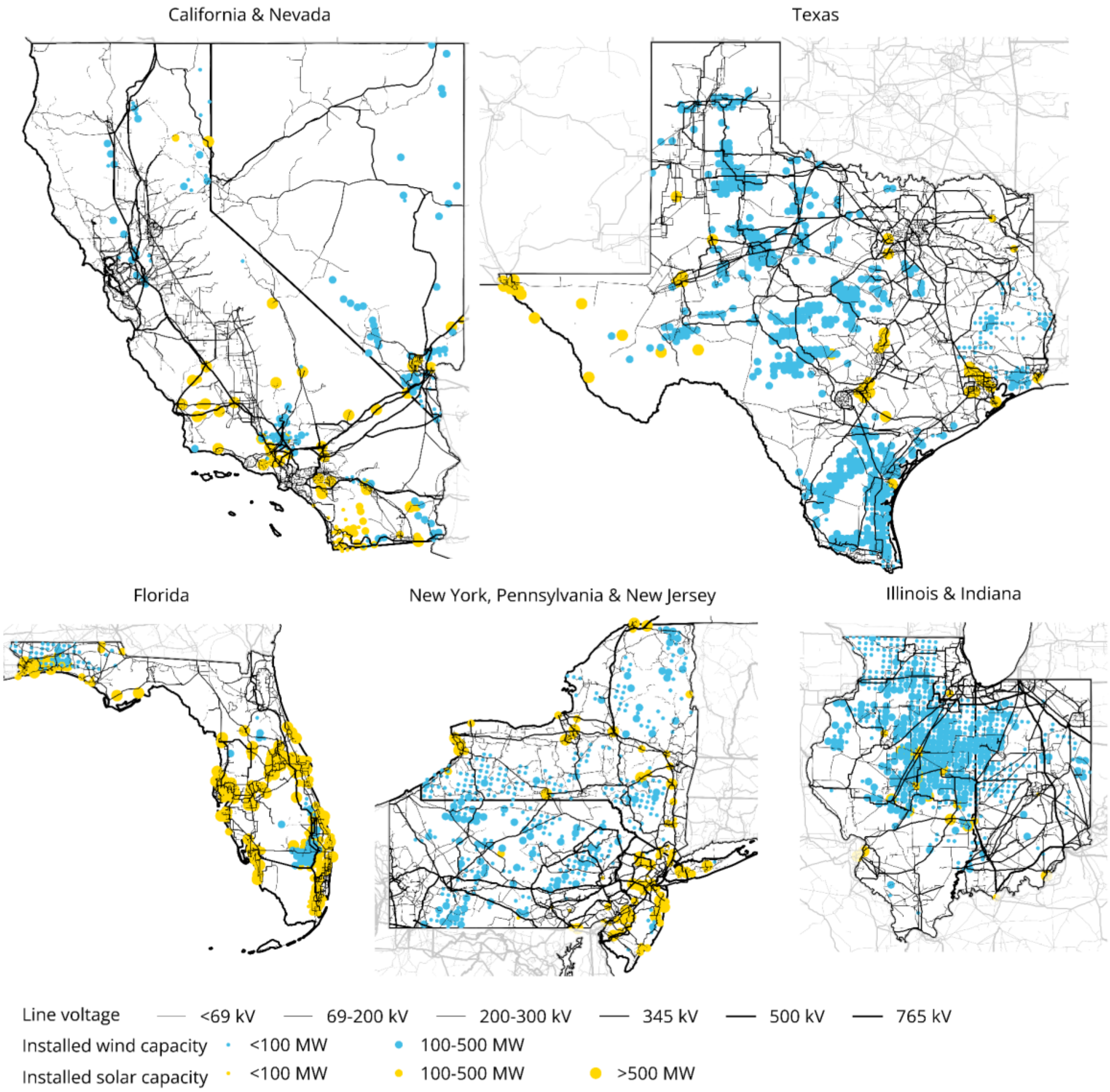

Fig. S5: Selected sites of solar and wind plants by the ReEDS model for the restricted build-out scenario with reconductoring are in close proximity to the existing US transmission network. Dots denote the locations of solar plants (in yellow) and wind farms (in blue) installed by 2035 for the restricted build-out scenario with reconductoring, underlaid with the existing US transmission network *(49)* for select states.

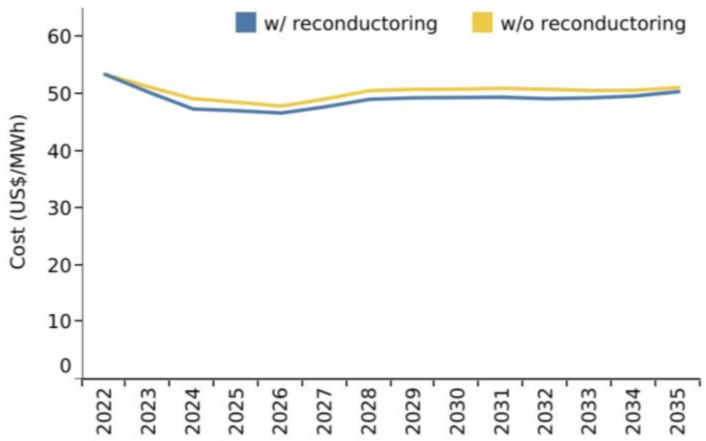

Fig. S6: Progression of wholesale electricity costs for the restricted build-out scenario. The combined effect of lower transmission expansion costs and higher-quality RE lowers wholesale electricity costs (the sum of transmission and generation costs, in US$/MWh) by 3-4% versus when reconductoring is not offered as an option.

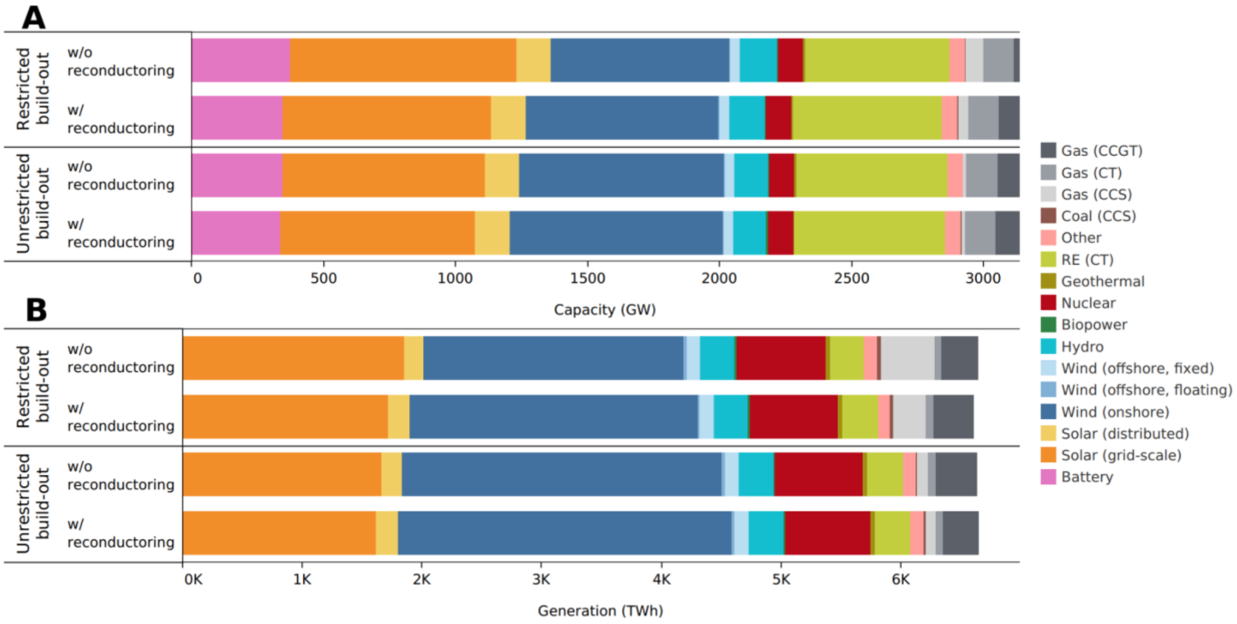

Fig. S7: Technological breakdown of installed capacity and annual generation US-wide in 2035. (A) Installed capacity. (B) Annual generation. The generation of battery capacity is included within the battery charging technology.

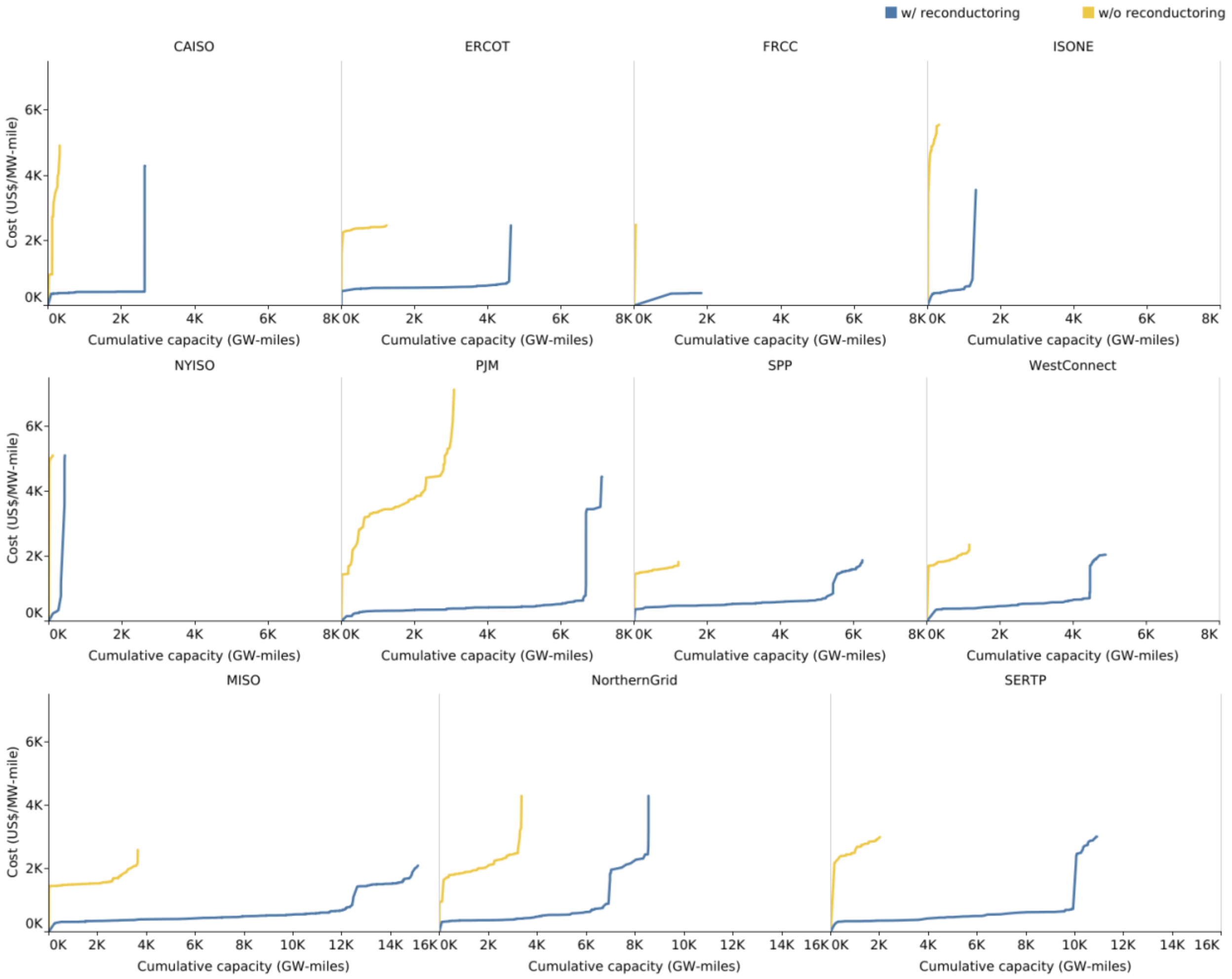

Fig. S8: Supply curves for adding transmission capacity by transmission region for the restricted build-out scenario. When reconductoring is offered as an option, the model builds more transmission capacity and at lower unit costs in both the unrestricted and restricted build-out scenarios. Supply curves were created by sorting the ReEDS paths' unit costs of reconductoring in ascending order and plotted as a function of cumulative GW-miles.

Table S1. Comparison of conventional conductors with different types of advanced conductors[1].

Abbr.	Full name	Year invented	Composition	Ampacity increase over ACSR	Operating temperature	"Drake"-size area[2]	"Drake"-size strength[3]	"Drake"-size weight[3]	"Drake"-size AC resistance at 75°C	Proprietary?	Deployment	Source(s)
ACSR	Aluminum Conductor Steel Reinforced	1900s	1350-H19 aluminum round strands around a galvanized steel core	1x	75°C cont., 100°C emerg.	795 kcmil	31,500 lbs	1093 lbs/kft	0.0263 Ohms/kft	no	En masse	*(2)*
ACSS	Aluminum Conductor Steel Supported	1970s	1350-O fully annealed aluminum round strands around a galvanized steel core	<1.7x	250°C cont./emerg.	795 kcmil	25,900 lbs	1093 lbs/kft[4]	0.0257 Ohms/kft	no	En masse	*(3)*
ACCR	Aluminum Conductor Composite Reinforced		Zirconium-doped aluminum trapezoidal strands around a core of aluminum oxide fibers embedded in an aluminum matrix	~2x	210°C cont., 240°C emerg.	958 kcmil	37,000 lbs	1075 lbs/kft	0.0213 Ohms/kft	yes: 3M	>6,000 miles installed on >325 projects	*(5,6)*
ACCS	Aluminum Conductor Composite Supported[5]		1350-O fully annealed aluminum trapezoidal strands around a composite core of carbon fiber in a polymer matrix	~2x	180°C cont., 225°C emerg.	995 kcmil	33,500 lbs	1001 lbs/kft	0.0290 Ohms/kft[6]	yes: Southwire	n/a	*(50)*
HVCRC	High Voltage Composite Reinforced Conductors	2000s	1350-O fully annealed aluminum trapezoidal strands around a glass-carbon fiber hybrid core	~2x	180°C cont., 200°C emerg.	1036 kcmil	43,000 lbs	1063 lbs/kft	0.0204 Ohms/kft	yes: Epsilon Cable	>4,500 miles in 10+ countries	*(51)*
ACCC	Aluminum Conductor Composite Core[7]		1350-O fully annealed aluminum trapezoidal strands around a composite core of carbon fiber encased in a fiberglass tube	~2x	180°C cont., 200°C emerg.	1026 kcmil	41,200 lbs	1052 lbs/kft	0.0202 Ohms/kft	yes: CTC Global	>100,000 miles installed on >1,250 projects in 60+ countries	*(7)*
AECC	Aluminum Encapsulated Carbon Core		1350-O fully annealed aluminum trapezoidal strands around a composite core of carbon fiber encapsulated in aluminum	~2-3x	180°C cont., 200°C emerg.	1051 kcmil	42,200 lbs	1050 lbs/kft	0.0199 Ohms/kft	yes: TS Conductor	>300 miles	*(8)*

[1] Technical conductor specifications are taken from manufacturers' data sheets and may vary slightly as a result of different environmental parameters at which measurements were taken. Further, we compare the standard conductor models, although manufacturers typically offer a portfolio of designs that may incorporate higher strength cores, different coatings and/or different aluminum alloys for different performance characteristics.

[2] Conductor size is measured in units of thousand circular mils (kcmil), where one circular mil equals the area of a circle with a diameter of one mil (a thousandth of an inch). We display characteristics for the "Drake"-size conductor, similar trends hold across different conductor sizes.

[3] Advanced conductors typically utilize a trapezoidal shape of the outer aluminum wires, in contrast to the more common round-wire construction of conventional conductors. The trapezoidal shape incorporates more aluminum, meaning strength and weight values for ACCR, ACCC and AECC may be slightly elevated compared to ACSR and ACSS.

[4] Standard strength, although high strength options are also available (*3*).

[5] Also known as the C^7 overhead conductor.

[6] AC resistance at 180°C since AC resistance at 75°C was not available.

[7] Other models include the ACCC AZR conductor (using an aluminum-zirconium alloy for greater strength) and the ACCC ULS conductor (incorporating more carbon fiber for ultra-low sag properties.

Table S2. Empirical project data.

Line	Transmission region	Project type	Commissioning year	Voltage	Line length	Original capacity	New capacity	Capex[1,2] (million US$)	Cost (million US$/mile)	Cost (million US$/GW-mile)	Source(s)
Avelin-Avelgem-Horta	Belgium	Reconductor	2022	380 kV	48 miles	3 GW	6 GW	193	4.0	1.3	*(21)*
Massenhoven-Van Eyck			2026		56 miles	3 GW	6 GW	140	2.5	0.8	*(21)*
Van Eyck-Gramme			2029		54 miles	3 GW	6 GW	164	3.0	1.0	*(21)*
Gramme-Courcelles			2033		43 miles	3 GW	6 GW	175	4.1	1.4	*(21)*
Courcelles-Bruegel			2035		29 miles	3 GW	6 GW	129	4.4	1.5	*(21)*
Bruegel-Mercator			2025		20 miles	3 GW	6 GW	93	4.7	1.6	*(21)*
Mercator-Massenhoven			2030		22 miles	3 GW	6 GW	82	3.8	1.3	*(21)*
Avelgem-Courcelles ("Boucle de Hainaut")[3]		New-build	2028		62 miles	-	6 GW	584	9.4	1.6	*(21)*
Stevin-Avelgem ("Ventilus")[3]			2028		56 miles	-	6 GW	467	8.4	1.4	*(21)*
Diemen-Lelystad-Ens	Netherlands	Reconductor	2022	380 kV	45 miles	3.3 GW[4]	5.3 GW[4]	123	2.8	1.4	*(21)*
Ens-Zwolle			2024		20 miles	3.3 GW[4]	5.3 GW[4]	48	2.4	1.2	*(21)*
Krimpen-Geertruidenberg			2023		21 miles	3.3 GW[4]	5.3 GW[4]	48	2.3	1.2	*(21)*
Eindhoven-Maasbracht			2025		30 miles	3.3 GW[4]	5.3 GW[4]	58	1.9	1.0	*(21)*
Zwolle-Hengelo-Doetinchem-Dodewaard			2040		102 miles	3.3 GW[4]	5.3 GW[4]	245	2.4	1.2	*(21)*
Borssele-Rilland ("ZuidWest380 West")		New	n/a		30 miles	-	-	659	22.1	-	*(21)*
Rilland-Tilburg ("ZuidWest380 Oost")			n/a		50 miles	-	-	1273	25.0	-	*(21)*
Zandvliet-Lillo-Liefkenshoek ("Brabo II")			2025		11 miles	-	-	117	10.5	-	*(21)*
Lower Rio Grande Valley	ERCOT	Reconductor	2016	345 kV	240 miles	1.2 GW[5]	2.4 GW[5]	225	0.9	0.7	*(31)*
CREZ lines		New	2013		3600 miles	-	-	6900	1.9[3]	2.5[3]	*(30)*
Bob-Mead	CAISO	Reconductor	n/a	230 kV	15 miles	-	n/a	25	1.7	-	*(53)*
Big Creek Corridor			2018		69 miles	-	n/a	6	0.1	-	*(52)*
Beatty		New	n/a		62 miles	-	-	155	2.5	-	*(54)*
Generic double circuit	MISO	Reconductor	-	345 kV	-	-	n/a	-	1.1	-	*(55)*
Generic double circuit		New	-		-	-	-	-	5.4	-	*(55)*

[1] Where applicable, Euros are converted to US$ with a conversion rate of 1.168 US$/EUR, the 10-year average between 2013-2022.

[2] Costs for new-build projects may be overly optimistic as they reflect planning costs for lines that were master planned and part of a larger transmission planning package.

[3] In Belgium, the costs of new-build projects reflect new lines which will be wired with ACCC.

[4] Capacity estimated based on provided current rating, voltage, number of circuits and other known information.

[5] CREZ lines' cost in US$/mile is calculated as the total capex over the summed line lengths, while the cost in US$/GW-mile is an average over all voltage levels.

Table S3. Reference conductor selection[1].

AC voltage range	ROW width	Surge Impedance (SI)	Surge Impedance Loading (SIL)	Bundle quantity[2]	Original conductor[3]	Upgraded conductor[3]
100-161 kV	120 ft	380 Ohms	26-68 MW	1	ACSR “Drake” (795 kcmil)	ACCC “Drake” (1026 kcmil)
220-287 kV	150 ft	375 Ohms	129-220 MW	1	ACSR “Bittern” (1272 kcmil)	ACCC “Bittern” (1582 kcmil)
345 kV	160 ft	366 Ohms	325 MW	2	ACSR “Drake” (795 kcmil)	ACCC “Drake” (1026 kcmil)
500 kV	180 ft	294 Ohms	850 MW	3	ACSR “Cardinal” (954 kcmil)	ACCC “Cardinal” (1222 kcmil)
765 kV	200 ft	266 Ohms	2200 MW	5	ACSR “Drake” (795 kcmil)	ACCC “Drake” (1026 kcmil)

[1] Based on *(55,56)*. Actual line configurations may vary by transmission planning region, the age of the line, etc.
[2] Bundle quantity is assumed to be the same for the original and upgraded conductor, yet bundle quantity may be modified within a reconductoring project depending on the project needs, structure capabilities, etc.
[3] Conductor size is measured in units of thousand circular mils (kcmil), where one circular mil equals the area of a circle with a diameter of one mil (a thousandth of an inch).

References

1. J. Caspary, J. Schneider, "Advanced Conductors on Existing Transmission Corridors to Accelerate Low Cost Decarbonization" (Grid Strategies LLC, 2022). https://acore.org/wp-content/uploads/2022/03/Advanced_Conductors_to_Accelerate_Grid_Decarbonization.pdf
2. "ACSR" (Southwire, 2019). https://www.southwire.com/wire-cable/bare-aluminum-overhead-transmission-distribution/acsr/p/ALBARE6
3. "ACSS" (Southwire, 2015). https://www.southwire.com/wire-cable/bare-aluminum-overhead-transmission-distribution/acss/p/ALBARE7
4. D. Bryant, "Why High-Capacity, Low-Sag ACCC Conductor Offers a Better Solution than ACSS" (CTC Global, 2018). https://ctcglobal.com/high-capacity-low-sag-accc-conductor-offers-better-solution-acss/
5. 3M Aluminum Conductor Composite Reinforced (ACCR): High-capacity transmission conductor" (3M, 2014). https://multimedia.3m.com/mws/media/478270O/3mtm-accr-high-capacity-transmission-conductor.pdf
6. "3M ACCR: More Amps, More Confidence" (3M, 2014). https://www.ieee.hr/_download/repository/Allan_Russell_3M_ACCR_%28Aluminum_Conductor_Composite_Reinforced%29_-_Proven_Solutions_to_Increase_Capacity.pdf
7. "ACCC Conductor Data Sheets" (CTC Global, n.d.). https://ctcglobal.com/accc-conductor-data-sheets/
8. "TS Conductor" (TS Conductor, 2023). https://tsconductor.com/products/
9. Leveraging Existing Infrastructure: Increasing the Capacity of Transmission Lines and Rights of Way" (Electric Power Research Institute, 2021). https://www.epri.com/research/products/000000003002023004
10. "Engineering Transmission Lines with High Capacity Low Sag ACCC Conductors" (CTC Global, 2011). https://documents.dps.ny.gov/search/Home/ViewDoc/Find?id=%7B4B3A8A12-3090-40E7-841F-EC1BEED0C95B%7D&ext=pdf
11. "IEEE Standard 738-2006 for Calculating the Current-Temperature of Bare Overhead Conductors" (IEEE, 2007). doi: 10.1109/IEEESTD.2007.301349
12. R. Pletka, J. Khangura, A. Rawlins, E. Waldren, D. Wilson, "Capital Costs for Transmission and Substations: Updated Recommendations for WECC Transmission Expansion Planning" (Black and Veatch, 2014). https://efis.psc.mo.gov/mpsc/commoncomponents/viewdocument.asp?DocId=936076825
13. J.F. Goffinet, "Elia Addresses the Need for More Capacity in Belgium" (T&D World, 2017). https://www.tdworld.com/overhead-transmission/article/20970140/elia-addresses-the-need-for-more-capacity-in-belgium
14. "Project at Belgian TSO Navigated Challenges Facing New Power Infrastructure" (INMR, 2020). https://www.inmr.com/project-belgian-tso-navigated-challenges-facing-new-power-infrastructure-2/
15. J.F Goffinet, "Elia Tackles Grid Reliability Through New Technologies" (T&D World, 2022). https://www.tdworld.com/transmission-reliability/article/21247687/elia-tackles-grid-reliability-through-new-technologies
16. "Federal Development Plan 2024-2034" (Elia, 2023). https://www.elia.be/en/infrastructure-and-projects/investment-plan/federal-development-plan-2024-2034
17. "Projects of Common Interest" (European Commission, n.d.). https://energy.ec.europa.eu/topics/infrastructure/projects-common-interest_en
18. "Beyond State-of-the-Art Technologies for Power AC Corridors and Multi-terminal HVDC Systems" (CORDIS, 2014). https://cordis.europa.eu/project/id/612748
19. "Directive 2009/72/EC of the European Parliament and of the Council concerning common rules for the internal market of electricity" (Official Journal of the European Union, 2009). https://faolex.fao.org/docs/pdf/eur124471.pdf
20. "Besluit tot vaststelling van de tariefmethodologie voor het elektriciteitstransmissienet en voor de elektriciteitsnetten met een transmissiefunctie voor de regulatoire periode 2024-2027" (CREG, 2022). https://www.creg.be/sites/default/files/assets/Publications/Others/Z1109-11NL.pdf
21. "TYNDP 2020 Project Sheets" (ENTSO-E, 2020). https://tyndp2020-project-platform.azurewebsites.net/projectsheets
22. "Beter Benutten Bestaande hoogspanningsverbindingen (380 kV) opwaarderen" (TenneT, 2022). https://www.tennet.eu/nl/projecten/beter-benutten-bestaande-380kv
23. "Studies on congestion management" (TenneT, 2022). https://www.tennet.eu/de-elektriciteitsmarkt/congestiemanagement/studies-congestion-management

24. "Regulation (EU) 2019/943 of the European Parliament and of the Council of 5 June 2019 on the internal market for electricity" (Official Journal of the European Union, 2019). https://eur-lex.europa.eu/legal-content/EN/TXT/?uri=CELEX%3A32019R0943
25. "Action plan of the Netherlands: Implementation of Articles 14, 15 & 16 of Regulation (EU) 2019/943" (Ministry of Economic Affairs and Climate Policy of the Netherlands, 2019). https://energeia-binary-external-prod.imgix.net/BBN6GLBKKXz_lH4ydtunYPfcpgc.pdf?dl=Actieplan+zone-overschrijdende+transportcapaciteit+elektriciteitshandel.pdf
26. "Terna: 2023 Development Plan for the National Electricity Grid Presented" (Terna, 2023). https://www.terna.it/en/media/press-releases/detail/2023-development-plan
27. "2023: The Hypergrid Project and Development Requirements" (Terna, 2023). https://download.terna.it/terna/2023_Hypergrid_project_and_development_requirements_8db79602cedc732.pdf
28. "Record Energized Reconductor Project Brings Reliable Power to South Texas" (EEI Energy Biz, 2016). https://www.quantaenergized.com/wp-content/uploads/2015/05/EEI-Energy-Biz_pages.pdf
29. "Board of Directors Meeting: September 20, 2011" (ERCOT, 2011). https://www.ercot.com/calendar/09202011-Board-of-Directors-Meeting
30. J. Warren, "Texas Million-Dollar Miles" (Concept Elemental, 2023). https://www.conceptelemental.com/commentary/2023/1/21/texas-million-dollar-miles
31. "Transmission line approval recommended by ERCOT board" (Power Engineering, 2011). https://www.power-eng.com/news/transmission-line-approval-recommended-by-ercot-board/#gref
32. "Manual on Transmission Planning Criteria" (Central Electricity Authority, 2023). https://cea.nic.in/wp-content/uploads/psp___a_ii/2023/03/Manual_on_Transmission_Planning_Criteria_2023.pdf
33. V. Pillai, "Our Outlook For India Remains Bullish: CTC Global" (T&D India, 2021). https://www.tndindia.com/our-outlook-for-india-remains-bullish-ctc-global/
34. "ACCC Conductor Installations: Project Map" (CTC Global, n.d.). https://ctcglobal.com/project-map/
35. V. Pillai, "In conversation with J.D. Sitton, CEO of CTC Global Corporation" (T&D India, 2019). https://ctcglobal.com/wp-content/uploads/T-and-D-India-March-2019.pdf
36. "An Evolving Market: Trends and growth drivers in the cables and conductors industry" (Powerline, 2018). https://powerline.net.in/2018/01/06/an-evolving-market/
37. "ACCR Should Be A Solution Of Choice To Transmission Utilities: 3M India" (T&D India, 2020). https://www.tndindia.com/accr-solution-choice-transmission-utilities-3m-india/
38. "CTC Global Joins Forces with World's Largest Electric Utility to Produce ACCC Conductor Core in China" (Business Wire, 2013). https://www.businesswire.com/news/home/20130814005174/en/CTC-Global-Joins-Forces-with-World%E2%80%99s-Largest-Electric-Utility-to-Produce-ACCC-Conductor-Core-in-China
39. H. Sok, "The Will to Power" (Global Trade Magazine, 2014). https://www.globaltrademag.com/the-will-to-power
40. D. Bryant, "ACCC Conductor Installed on Milestone 1100 kV DC Project in China" (CTC Global, 2020). https://ctcglobal.com/accc-conductor-installed-on-milestone-1100-kv-dc-project-in-china/
41. D. S. Carvalho Jr. et al., "Sectionalizing transmission lines, an expansion planning challenge, amplified by unexpected emerging variable renewable generation and environmental restrictions" (CIGRE, 2022). https://www.epe.gov.br/sites-pt/publicacoes-dados-abertos/publicacoes/PublicacoesArquivos/publicacao-725/topico-670/C1_-_Full_Papers_PS1_10156_2022.pdf
42. 2022-2023 Transmission Plan. (California Independent System Operator, 2023). http://www.caiso.com/InitiativeDocuments/ISO-Board-Approved-2022-2023-Transmission-Plan.pdf
43. "Greenlink Nevada" (NV Energy, 2021). https://lands.nv.gov/uploads/meeting_minutes/E2021-098.pdf
44. "Power Flow Test Systems Repository" (Al-Roomi, 2015). http://www.al-roomi.org/power-flow/3-bus-systems/system-iii
45. R. D. Zimmerman, C. E. Murillo-Sanchez, "Matpower User's Manual, Version 7.1" (Power Systems Engineering Research Center, 2020). https://matpower.org/docs/MATPOWER-manual.pdf
46. D. Lauria, F. Mottola, S. Quaia, Analytical Description of Overhead Transmission Lines Loadability. *Energies* **12**, 3119 (2019). https://doi.org/10.3390/en12163119
47. D. Lauria, S. Quaia, "An investigation on line loadability increase with high temperature conductors" in *2017 6th International Conference on Clean Electrical Power (ICCEP)* (IEEE, Santa Margherita Ligure, Italy, 2017), pp. 645-649. https://doi.org/10.1109/ICCEP.2017.8004757

48. R. Gutman, P.P. Marchenko, R.D. Dunlop, Analytical Development of Loadability Characteristics for EHV and UHV Transmission Lines. *IEEE Transactions on Power Apparatus and Systems* **PAS-98** (2) (1979). https://doi.org/10.1109/TPAS.1979.319410
49. Data from "Transmission Lines, United States Homeland Infrastructure Foundation Level Database (HIFLD)". Available at https://hifld-geoplatform.hub.arcgis.com/datasets/geoplatform::transmission-lines/about. Deposited 15 December 2022.
50. "C7 overhead conductor" (Southwire, 2017). https://overheadtransmission.southwire.com/wp-content/uploads/2017/06/1904_C7-Brochure_IMPERIAL_WEB_SPREADS.pdf
51. "HVCRC Data Sheets" (Epsilon Cable, 2023). https://www.epsilon-cable.com/resources/hvcrc-data-sheets
52. "Big Creek Corridor Rating Increase" (Southern California Edison, 2016). https://www.caiso.com/Documents/SCEPresenation-2016-2017TransmissionPlanningProcess.pdf
53. "Board-Approved 2017-2018 Transmission Plan" (CAISO, 2018). https://efiling.energy.ca.gov/GetDocument.aspx?tn=223248&DocumentContentId=20064
54. "Appendix F: Detailed Policy Assessment of the 2022-2023 Transmission Plan" (CAISO, 2023). http://www.caiso.com/InitiativeDocuments/Appendix-F-Revised-Draft-2022-2023-Transmission-Plan.pdf
55. "Transmission Cost Estimation Guide" (MISO, 2022). https://cdn.misoenergy.org/20220208%20PSC%20Item%2005c%20Transmission%20Cost%20Estimation%20Guide%20for%20MTEP22_Draft622733.pdf
56. M. Goldberg, D. Keyser, "Transmission Line Jobs and Economic Development Impact (JEDI) Model User Reference Guide" (National Renewable Energy Laboratory, 2013). https://www.nrel.gov/docs/fy14osti/60250.pdf
